# A Comparative Study of *C*_2_-Symmetric and *C*_1_-Symmetric Hydroxamic Acids in Vanadium-Catalyzed Asymmetric Epoxidation of Allylic Alcohols

**DOI:** 10.3390/molecules30214311

**Published:** 2025-11-06

**Authors:** Marco Valtierra-Galván, Alfredo Rodríguez-Hernández, Israel Bonilla-Landa, Felipe Barrera-Méndez, Francisco Javier Enríquez-Medrano, Ramón Enrique Díaz de León-Gómez, José Luis Olivares-Romero

**Affiliations:** 1 Red de Estudios Moleculares Avanzados, Campus III, Instituto de Ecología, A. C., Carretera Antigua a Coatepec 351, Xalapa 91073, Mexico; marco.valtierra@posgrado.ecologia.edu.mx (M.V.-G.); zs22000708@estudiantes.uv.mx (A.R.-H.); israel.bonilla@inecol.mx (I.B.-L.); felipe.barrera@inecol.mx (F.B.-M.); 2 Research Center in Applied Chemistry (CIQA), Enrique Reyna Hermosillo, No. 140. Col. San José de los Cerritos, Saltillo 25294, Mexico; javier.enriquez@ciqa.edu.mx (F.J.E.-M.); ramon.diazdeleon@ciqa.edu.mx (R.E.D.d.L.-G.)

**Keywords:** asymmetric epoxidation, hydroxamic acids, vanadium catalysis, ligand symmetry, enantioselectivity, *C*_1_/*C*_2_ ligands

## Abstract

Hydroxamic acids are emerging as versatile chiral ligands for metal-catalyzed asymmetric oxidations due to their tunable electronic and steric environments. In this study, we systematically compared the catalytic behavior of *C*_2_- and *C*_1_-symmetric hydroxamic acid ligands in the vanadium-catalyzed asymmetric epoxidation of allylic alcohols. A series of chiral hydroxamic acids (HA1–HA7) was synthesized and evaluated under varied conditions to elucidate the influence of ligand symmetry on enantioinduction and reactivity. The results demonstrate that *C*_2_-symmetric bishydroxamic acids generate a highly organized chiral environment, leading to high enantioselectivity but often limited conversion, consistent with the Sabatier principle. Conversely, certain *C*_1_-symmetric ligands—particularly HA3—produced notable enantioselectivity (up to 71% *e.e.*) and full conversion under optimized conditions with VO(O*i*Pr)_3_ in CH_2_Cl_2_. A quadrant-based stereochemical model is proposed to rationalize the differential performance of these ligands. These findings highlight the critical role of ligand desymmetrization in modulating the chiral environment around vanadium centers, providing valuable design principles for next-generation hydroxamic acid-based catalysts in asymmetric synthesis. The optimized system (VO(O*i*Pr)_3_/HA3 in CH_2_Cl_2_) afforded >99% conversion and 71% *e.e.*, providing a basis for extending hydroxamic acid scaffolds to diverse allylic alcohols.

## 1. Introduction

Developing efficient and selective catalysts for asymmetric epoxidation reactions remains a central challenge in organic synthesis, as these transformations provide access to chiral building blocks with broad synthetic utility. Among the various catalytic systems explored, hydroxamic acids (HAs) have attracted considerable attention due to their ability to form stable metal complexes and promote highly enantioselective oxidations [1,2]. In recent years, hydroxamic acid ligands have been widely investigated in asymmetric catalysis, particularly for metal-mediated oxidation reactions, where they enable fine control of both activity and selectivity [3,4]. Their modular structure allows systematic tuning of steric and electronic properties, making them versatile scaffolds for designing novel asymmetric catalysts [5,6]. Early studies demonstrated that vanadium and titanium complexes bearing chiral hydroxamic acids could catalyze the enantioselective epoxidation of allylic alcohols, offering an appealing alternative to classical Sharpless and Jacobsen systems [7,8,9]. These investigations established that the coordination environment of the metal center—defined by the symmetry, rigidity, and donor characteristics of the ligand—plays a decisive role in enantioinduction. Subsequent developments introduced C_2_-symmetric bishydroxamic acids (BHAs) to reinforce the chiral pocket and minimize conformational freedom, often achieving excellent enantioselectivities but sometimes at the expense of catalytic turnover or substrate scope [10]. The observation of strong enantioselectivity but low conversion in vanadium- and titanium-catalyzed systems can be rationalized by the Sabatier principle, which dictates that optimal catalytic efficiency arises from a balanced catalyst–substrate interaction [11]. Under certain conditions, particularly with rigid C_2_-symmetric ligands, the substrate binds too tightly to the metal center, stabilizing the chiral transition state but reducing overall turnover. Solvent polarity and temperature strongly modulate this balance: nonpolar media often favor selectivity, whereas polar or coordinating solvents enhance reactivity [12]. This interplay underscores the need for catalyst designs that harmonize selectivity and activity. As noted by Pfaltz and others, there is no intrinsic reason that C_2_ symmetry should universally outperform C_1_-symmetric or desymmetrized ligands [13]. Indeed, nonsymmetrical frameworks possessing electronically or sterically differentiated donor groups can generate asymmetric fields that rival or surpass those of symmetric analogues [14,15,16]. In this context, Yamamoto and Zhang (2007) emphasized that the rational design of chiral catalysts depends on achieving a balance between rigidity and adaptability within the chiral environment, highlighting that symmetry should be considered a tunable parameter rather than an absolute requirement for high enantioselectivity [17]. This conceptual shift provides the theoretical foundation for our comparative investigation.

Building upon these insights, the present study systematically evaluates C_2_-symmetric bishydroxamic acids (BHA1 and BHA2) and C_1_-symmetric hydroxamic acids (HA1–HA7) as ligands for vanadium-catalyzed asymmetric epoxidation of allylic alcohols. Conducted under standardized conditions, this work elucidates how ligand symmetry governs catalytic efficiency and stereochemical outcome. By comparing these two ligand families within a unified experimental framework, we aim to clarify the mechanistic relationship between symmetry, steric bias, and enantioselectivity, thereby contributing to the rational design of hydroxamic-acid-based catalysts for asymmetric oxidation reactions.

## 2. Results and Discussion

### 2.1. Synthesis of N-Obz Amines

**Scheme 1:**
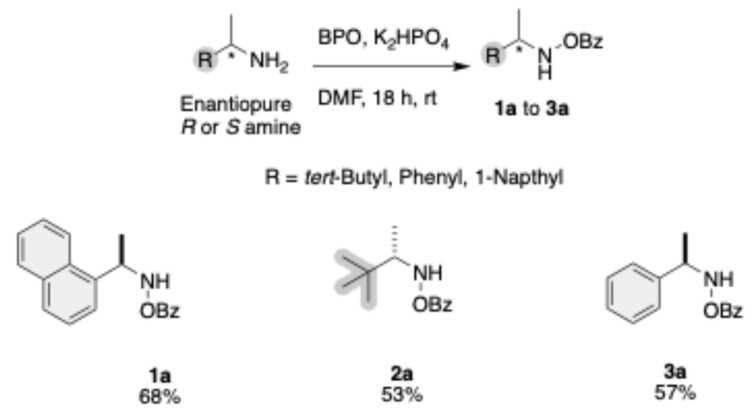
Synthesis of *N*-OBz amines.

The enantiopure amines were transformed to the corresponding *N*-O Benzoyl amines using BPO as oxidant, and potassium phosphate (K_2_HPO_4_) as a base in dimethylformamide (DMF) as the solvent. This reaction proceeds over 18 h at room temperature to yield the intermediates **1a**, **2a**, and **3a**, with yields up to 68% (Scheme 1). All intermediates in this pathway share a common benzoyl-protected (OBz) amine group.

**Scheme 2:**
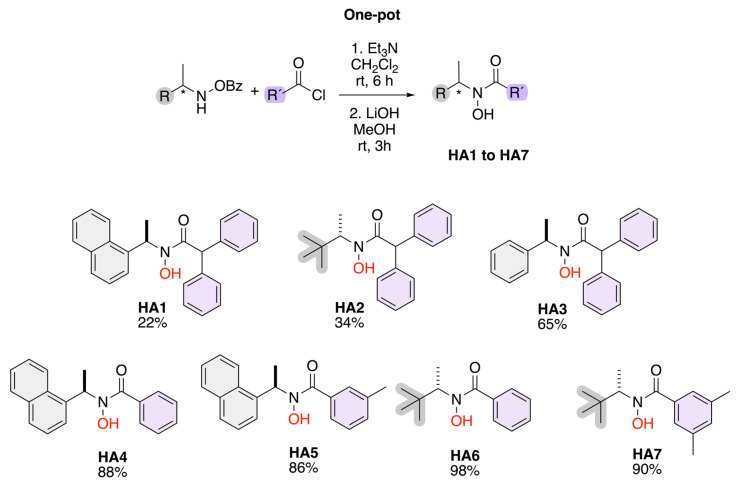
Synthesis of chiral hydroxamic acids.

Scheme 2 illustrates a one-pot synthetic route for the preparation of hydroxamic acids (HA1–HA7) from benzoyl-protected hydroxamic acid precursors. The reaction proceeds through a two-step process: first, acylation of the hydroxamic acid derivative with an appropriate acyl chloride (R′–COCl) in the presence of triethylamine (Et_3_N) in dichloromethane (CH_2_Cl_2_) at room temperature for 6 h; second, deprotection of the benzoyl group using lithium hydroxide (LiOH) in methanol (MeOH) at room temperature for 3 h. The resulting compounds (HA1–HA7) exhibit structural diversity in both R and R′ substituents, providing a versatile library of hydroxamic acids, with yields up to 98%.

We began our investigation into how ligand symmetry influences enantioinduction by comparing the performance of *C*_2_- versus *C*_1_-symmetric hydroxamic acid ligands (BHA1, BHA2, and HA3) in the vanadium-catalyzed epoxidation of allylic alcohol **8** (Table 1).

As shown in Table 1, the *C*_2_-symmetric ligands BHA1 and BHA2 provided low conversions (14–11%) and poor enantioselectivity (9–7% *e.e.*) even at low temperature (−20 °C), indicating that the highly rigid chiral environment imposed by the *C*_2_ framework restricts productive substrate binding. This result aligns with the Sabatier principle, suggesting that excessive stabilization of the metal–ligand–substrate complex hinders catalytic turnover. To our delight, the *C*_1_-symmetric ligand HA3 delivered full conversion (>99%) and markedly higher enantioselectivity (up to 71% *e.e.*) at room temperature. Guided by these preliminary results, the subsequent phase of this study focused on optimizing the epoxidation reaction under various conditions.

### 2.2. Analysis of Epoxidation Reaction Conditions

The epoxidation reactions were carried out using various chiral hydroxamic acids (HAs) and Lewis acids in toluene at room temperature to evaluate their impact on conversion and enantioselectivity. The results are summarized in the table below:

Table 2 presents the results of asymmetric epoxidation reactions conducted using different metal catalysts and hydroxy acids (HAs) in toluene at room temperature. The conversion and enantiomeric excess values are reported for each combination of metal and HA, providing insight into the catalytic efficiency and selectivity of the tested systems. These results reveal a significant disparity in the activity of the two metal catalysts used: Ti(O*i*Pr)_4_ and VO(O*i*Pr)_3_. When Ti(O*i*Pr)_4_ was combined with HA3 or HA2, as shown in Table 2, entries 2 and 5, the reaction did not proceed, resulting in no conversion. This outcome suggests that Ti(O*i*Pr)_4_ is not an effective catalyst for this epoxidation reaction under the given conditions. In contrast, VO(O*i*Pr)_3_ consistently achieved quantitative conversion across all entries where it was tested with various HAs (Table 2, entries 1, 3, 4, 6-10), indicating its effectiveness as a catalyst for promoting the epoxidation reaction in toluene at room temperature. The enantiomeric excess varied significantly depending on the HA used in conjunction with VO(O*i*Pr)_3_. HA3, as shown in Table 2, entry 6, produced an enantioselectivity of 19%, indicating a modest level of enantioselectivity. HA2, Table 2 in entry 3, resulted in a slightly higher enantiomeric excess of 22%, suggesting that this ligand might provide slightly better chiral induction than HA3 under these conditions, possibly due to the incorporation of the *tert*-butyl group. HA1, shown in Table 2, entry 1, afforded an enantioselectivity of 15%, which is lower than that obtained with HA2 and HA3, indicating reduced stereochemical induction. This result suggests that the planar naphthyl group provides limited steric differentiation around the metal center, thereby contributing poorly to the chiral environment necessary for achieving high enantioselectivity. Notably, HA6, Table 2, entry 9, showed a significantly lower enantiomeric excess of 4%, suggesting poor chiral induction. On the other hand, HA7, as presented in Table 2, entry 10, afforded the highest enantioselectivity of 49%, indicating that it is the most effective ligand for enantioselective epoxidation among those evaluated. The presence of substituents on the aromatic ring α to the carbonyl group appears to be crucial for enhancing steric differentiation and achieving higher enantioselectivity. HA4, in Table 2, entry 7, resulted in an enantiomeric excess of 11%, showing moderate effectiveness in inducing chirality, while HA5, shown in Table 2, entry 8, produced an enantioselectivity of 30%, which is relatively high compared to most other HAs but still lower than HA7. Again, the substituents on the aromatic ring α to the carbonyl group play a vital role in controlling the stereoselectivity of the epoxidation.

Table 2, entry 4, presents the results using VO(acac)_2_ as the metal catalyst with HA2. The conversion remains quantitative, but the enantioselectivity is 19%, which is comparable to the results obtained with VO(O*i*Pr)_3_ and HA3 (Table 2, entry 6). This similarity indicates that while VO(acac)_2_ is effective in achieving conversion, its enantioselectivity is like that of VO(O*i*Pr)_3_ under these conditions. In general, VO(O*i*Pr)_3_ proves to be a superior catalyst compared to Ti(O*i*Pr)_4_, achieving full conversion with all HAs tested, while Ti(O*i*Pr)_4_, under these conditions, fails to catalyze the reaction. The choice of HA significantly influences the enantioselectivity, with HA7 standing out as the most effective ligand, producing the highest enantiomeric excess of 49%. Other HAs show varying degrees of effectiveness, with some, like HA6, performing poorly in terms of enantiomeric excess. Despite these findings, the overall enantioselectivity achieved suggests that further optimization is needed. This could involve modifying the ligand structure, adjusting reaction conditions such as temperature or solvent, or exploring other metal catalysts to improve the efficiency and selectivity of the catalytic system.

Based on previous results, we decided to analyze the solvent effect. As seen in Table 3, entries 1 to 4, we explored the impact of different solvents (CH_2_Cl_2_ and CH_3_CN) on the epoxidation reaction using HA2 and HA3 with VO(O*i*Pr)_3_. In CH_2_Cl_2_, HA2 resulted in 99% conversion with a moderate enantioselectivity of 23% (Table 3, entry 1). However, the same HA in CH_3_CN drastically reduced the enantioselectivity to 4% (Table 3, entry 2). HA3, on the other hand, exhibited significantly higher enantioselectivity in both solvents, with 71% in CH_2_Cl_2_ (Table 3, entry 3) and 63% in CH_3_CN (Table 3, entry 4). These results indicate that CH_2_Cl_2_ is generally a better solvent for achieving higher enantioselectivity with these HAs. Next, regarding the effect of catalyst loading, entries 5 and 6 in Table 3, examine the impact of reducing the catalyst and ligand loadings using VO(O*i*Pr)_3_ and HA2 in toluene. Lowering both the catalyst and the ligand loading to 5% mol and 7% mol, respectively, resulted in a decrease in enantioselectivity to 8% (Table 3, entry 5). However, maintaining the same conditions but with an increased reaction time of 48 h, the reaction showed a significant improvement in enantioselectivity to 55% (Table 3, entry 6), highlighting the importance of sufficient reaction time, catalyst, and ligand concentrations for optimal performance. Then, entries 8 and 9 in Table 3 investigate the effect of different additives on the reaction outcome with VO(O*i*Pr)_3_ and HA2 in toluene. Using molecular sieves (MS 4Å) and MgO as additives resulted in slight improvements in enantioselectivity, achieving 18% and 22%, respectively, (Table 3, entries 8 and 9). While these additives showed some positive effects, the improvements were not substantial compared to the reactions without additives. Additionally, entries 12 to 19 in Table 3 explore the influence of temperature on the reaction using various HAs in toluene. Lowering the temperature to 0 °C generally improved enantioselectivity slightly compared to room temperature reactions. For example, HA3 showed a 19% enantiomeric excess at room temperature (Table 2, entry 2) when the same experiment was run at 0 °C, the result was 23% enantiomeric excess (Table 3, entry 13). Similarly, HA5 resulted in 24% enantiomeric excess at 0 °C (Table 3, entry 18). Finally, the best-performing activity of 71% was observed with HA3 in CH_2_Cl_2_ at room temperature (Table 3, entry 3). This result suggests that HA3 in combination with VO(O*i*Pr)_3_ and CH_2_Cl_2_ is the optimal condition among those tested for achieving high enantioselectivity in the epoxidation reaction.

**Figure 1:**
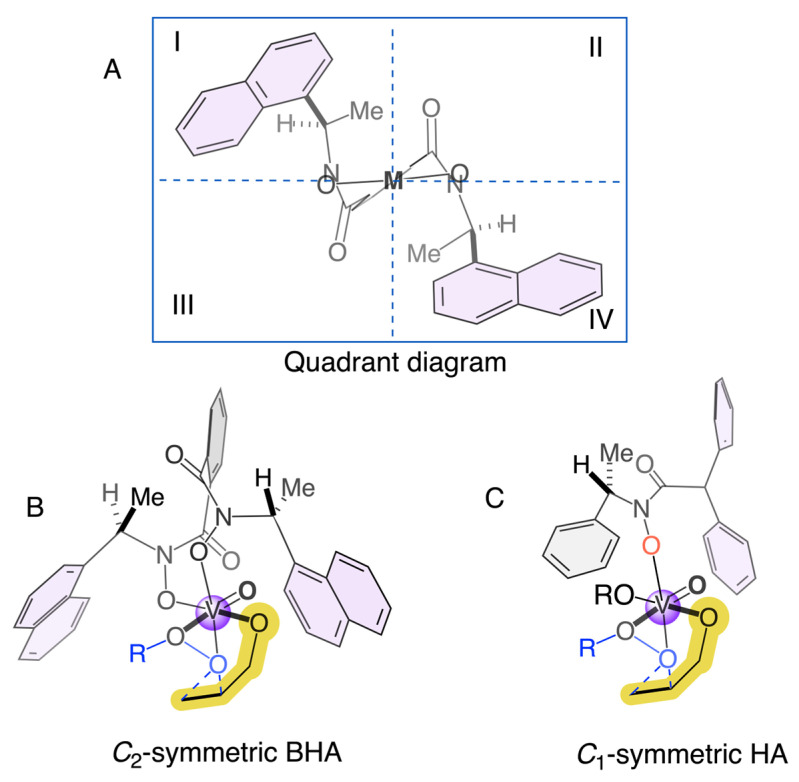
Plausible transition state of the *C*_2_-symmetric BHA (**B**) versus *C*_1_-HA (**C**) and quadrant diagram (**A**).

The discrepancies in enantioselectivity between Table 2 and Table 3 can be attributed primarily to the effects of solvent polarity and temperature on the chiral vanadium complex. In Table 2, reactions performed in toluene at room temperature yielded full conversions but moderate enantioselectivities, consistent with the limited stabilization of the asymmetric transition state in a nonpolar medium. In contrast, the results in Table 3 demonstrate that moderately polar solvents such as CH_2_Cl_2_ enhance enantioinduction by improving coordination and transition-state stabilization. The lower enantioselectivity observed with HA7 at 0 °C (Table 3, entries 11 and 16) suggests that reduced molecular mobility at low temperature hampers the formation of the most favorable chiral arrangement. Overall, these findings suggest that solvent coordination, ligand asymmetry, and temperature act synergistically to determine enantioselectivity, with optimal results obtained for VO(O*i*Pr)_3_/HA3 in CH_2_Cl_2_ at room temperature (71% *e.e.*). This solvent- and temperature-dependent behavior aligns well with the quadrant-based stereochemical model proposed in Figure 1, where the interplay between ligand geometry and the electronic environment around vanadium defines the extent of asymmetric induction.

Finally, Figure 1 provides a visual rationale for the enantioselectivity trends observed in our study of hydroxamic acid ligands. The Quadrant diagram (Section A) illustrates the chiral environment around the vanadium center using a quadrant model, where the (*R*,*R*)-ligand’s architecture defines equivalently sterically and electronically regions II and III. The *C*_2_-symmetric bishydroxamic acid (Section B) exhibits the superior performance typically associated with *C*_2_-symmetric frameworks, as its inherent symmetry generates a well-defined and rigid chiral pocket that consistently promotes one enantiofacial approach of the allylic alcohol substrate.

In contrast, the *C*_1_-symmetric hydroxamic acids (HAs) investigated in this work introduce structural asymmetry, leading to a less uniform and more flexible chiral environment. As depicted in Figure 1, section C, the non-identical substituents in a *C*_1_-symmetric ligand result in a less symmetrical arrangement of the quadrants around the metal center. This flexibility can lead to a distribution of substrate-catalyst conformations, which often diminishes enantiocontrol, as reflected in the generally moderate enantiomeric excess values obtained. Nevertheless, the exceptional case of HA3, which achieved 71% *e.e.*, suggests that with precise steric and electronic tuning, the unique geometry of a *C*_1_-symmetric ligand can create a highly selective chiral pocket that rivals or even surpasses the performance of more symmetrical analogs, underscoring the potential for further optimization in ligand design. The quadrant representation in Figure 1 is intended as a working stereochemical model that rationalizes the observed trends in enantioselectivity for HA1–HA7 under the conditions studied. Although this model captures the influence of steric bias and desymmetrization around vanadium, its broader validity across different substrates and oxidants remains to be established. Ongoing DFT calculations and expanded substrate screening are being pursued to assess its predictive value.

## 3. Materials and Methods

**Synthesis of Chiral BHA’s and HA’s**

The BHA1 and BHA2 were synthesized following previous literature reports [18]. In the case of the HA’s, we use the methodology reported by Yamamoto [19], the chiral hydroxylamines were obtained through oxidation with Benzoyl peroxide (BPO) of commercially available enantioenriched amines, sourced from Sigma–Aldrich (Burlington, Massachusetts, United States), then the hydroxamic acids (HA) were synthesized via a coupling reaction between Acyl chloride and chiral hydroxylamines. Further experimental procedures and NMR spectra are available in the Supplementary Materials.

**Synthesis of Synthesis of *N*-OBz amines (1a to 3a); General Procedure A**

A mixture of BPO (1.0 mmol) and K_2_HPO_3_ (1.5 mmol) in dimethyl formamide (DMF) was stirred at room temperature for 2 h. Then, a solution of enantiopure amine (0.5 mmol) in CH_2_Cl_2_ was added, and the solution was stirred for 18 h. Water was added, followed by extraction with CH_2_Cl_2_. The organic layer was washed with brine, dried over Na_2_SO_4_, and concentrated. The crude product was purified by silica gel column chromatography using a hexane/ethyl acetate (EtOAc) eluent system. The purified product was obtained after the solvent was evaporated [16].

Synthesis of **((*R*)-O-benzoyl-*N*-(1-(naphthalen-1-yl)ethyl)hydroxylamine) (1a).** General procedure A afforded the product 1a as a yellow oil in 68% yield. The product was purified by flash column chromatography (15% hexane/EtOAc). ^1^H NMR (500 MHz, CDCl_3_): δ = 8.20 (d, *J* = 8.4 Hz, 1H), 8.09 (s, 1H), 7.94 (d, *J* = 7.9 Hz, 2H), 7.81 (dd, *J* = 15.5, 7.7 Hz, 2H), 7.53 (dq, *J* = 14.6, 7.2 Hz, 4H), 7.41 (t, *J* = 7.6 Hz, 2H), 5.18 (q, *J* = 5 Hz, 1H), 1.69 (d, *J* = 10 Hz, 3H), 1.58 (s, 1H). ^13^C NMR (125 MHz, CDCl_3_) δ = 167.0, 136.7, 133.9, 133.4, 131.1, 129.54, 129.0, 128.5, 128.3, 126.3, 125.6, 123.7, 122.8, 56.3, 19.6. HRMS (ESI+): *m*/*z* [M + Na]^+^ calcd for C_19_H_17_NO_2_Na^+^: 314.1157; found: 314.1165.

Synthesis of **((*S*)-O-benzoyl-*N*-(3,3-dimethylbutan-2-yl)hydroxylamine) (2a).**

General procedure A afforded the product 2a as a colorless oil in 53% yield. The product was purified by flash column chromatography (20% hexane/EtOAc). ^1^H NMR (500 MHz, CDCl_3_) *δ* = 8.03 (d, *J* = 10, Hz, 2H), 7.59 (t, *J* = 5Hz, 1H), 7.46 (t, *J* = 5 Hz, 2H), 2.93 (q, *J* = 8.1, 5 Hz, 1H), 1.18 (d, *J* = 5 Hz, 3H), 1.05 (s, 9H). ^13^C NMR (125 MHz, CDCl_3_) *δ* = 167.5, 133.6, 129.6, 128.9, 65.4, 34.1, 27.1, 14.0. HRMS (ESI+): *m*/*z* [M + Na]^+^ calcd for C_13_H_19_NO_2_Na^+^: 244.1313; found: 244.1316.

Synthesis of **((*R*)-O-benzoyl-*N*-(1-phenylethyl)hydroxylamine) (3a).** General procedure A afforded the product 3a as a colorless oil in 57% yield. The product was purified by flash column chromatography (15% hexane/EtOAc). ^1^H NMR (500 MHz, CDCl_3_) δ = 8.12 (d, *J* = 8.1 Hz, 1H), 7.97 (d, *J* = 6.9 Hz, 2H), 7.69 (t, *J* = 7.5 Hz, 1H), 7.61–7.52 (m, 4H), 7.39 (dd, *J* = 8.4, 6.7 Hz, 2H), 7.33 (t, *J* = 7.2 Hz, 1H), 4.36 (q, *J* = 6.6 Hz, 1H), 1.57 (d, *J* = 6.7 Hz, 3H). HRMS (ESI+): *m*/*z* [M + Na]^+^ calcd for C_15_H_15_NO_2_Na: 264.1000; found: 264.1007

**Synthesis of Hydroxamic acids (H1-H7); General Procedure B**

Acyl chloride (1.0 mmol, 1.0 equiv) in CH_2_Cl_2_ and triethylamine (2.2 mmol, 2.2 equiv) were added to a solution of the *N*-(benzoyloxy)amine derivative (2.2 mmol, 2.2 equiv) in CH_2_Cl_2_. The resulting mixture was stirred for 6 h at room temperature. After the addition of water, the mixture was stirred for an additional 5 min and extracted with CH_2_Cl_2_ (2 × 10 mL). The combined organic extracts were concentrated under reduced pressure, and the residue was dissolved in methanol (CH_3_OH). LiOH·H_2_O (1.0 mmol, 1.0 equiv) was then added, and the reaction mixture was stirred under a nitrogen atmosphere for 10 min. The solvent was removed under reduced pressure, and the residue was treated with water, followed by extraction with CH_2_Cl_2_ (2 × 10 mL). The combined organic layers were washed sequentially with saturated NaHCO_3_ solution and brine, dried over anhydrous Na_2_SO_4_, and concentrated under reduced pressure to afford the crude product. Purification by silica gel column chromatography (hexane/EtOAc = 80:20) yielded the desired product after collecting the appropriate fractions and evaporating the solvent.

Synthesis of (**(*R*)-*N*-hydroxy-*N*-(1-(naphthalen-1-yl)ethyl)-2,2-diphenylacetamide**) (**HA1**). General procedure B afforded the product HA1 as a white solid in 22% yield. The product was purified by precipitating the product from a mixture of toluene or benzene and hexane at 0 °C. ^1^H NMR (500 MHZ, DMSO-*d*_6_): δ = 9.80 (*s*, 1 H), 8.17–8.08 (*m*, 1 H), 7.97–7.84 (*m*, 2 H), 7.50 (*ddt*, *J* = 32.4, 18.3, 8.8 Hz, 4 H), 7.37–7.15 (*m*, 10 H), 6.44 (*q*, *J* = 6.8 Hz, 1 H), 5.66 (*s*, 1 H), 1.58 (*d*, *J* = 6.8 Hz, 3 H). ^13^C NMR (125 MHz, DMSO-*d*_6_): δ = 171.3, 140.5, 140.4, 136.4, 133.8, 131.6, 129.4, 129.3, 129.0, 128.7, 128.5, 128.4, 128.0, 127.9, 127.1, 126.9, 126.6, 126.1, 125.7, 125.4, 123.8, 52.2, 49.9, 16.8. HRMS (ESI+): *m*/*z* [M + Na]^+^ calcd for C_26_H_23_NO_2_Na^+^: 404.1626; found: 404.1652

Synthesis of (**(*S*)-*N*-(3,3-dimethylbutan-2-yl)-*N*-hydroxy-2,2-diphenylacetamide**) (**HA2**). General procedure B afforded the product HA2 as a white solid in 34% yield. The product was purified by precipitating the product from a mixture of toluene or benzene and hexane at 0 °C. ^1^H NMR (500 MHZ, DMSO-*d*_6_): δ = 9.5 (br, 1H), 7.32–7.18 (*m*, 10 H), 5.71 (*s*, 1 H), 4.35 (*q*, *J* = 5 Hz, 1 H), 1.04 (*d*, *J* = 5 Hz, 3 H), 0.85 (*s*, 9 H). ^13^C NMR (125 MHz, DMSO-*d*_6_): δ = 171.6, 140.9, 129.4, 129.2, 128.6, 128.5, 126.9, 57.5, 51.9, 35.1, 27.5, 12.5. HRMS (ESI+): *m*/*z* [M + Na]^+^ calcd for C_20_H_25_NO_2_Na^+^: 334.1783; found: 334.1813.

Synthesis of (**(*R*)-*N*-hydroxy-2,2-diphenyl-*N*-(1-phenylethyl)acetamide**) (**HA3**). General procedure B afforded the product HA3 as a white solid in 65% yield. The product was purified by precipitating the product from a mixture of toluene or benzene and hexane at 0 °C.^1^H NMR (500 MHZ, DMSO-*d*_6_): δ = 7.37–7.14 (*m*, 15 H), 5.68 (*s*, 1 H), 5.67 (*q*, *J* = 10 Hz, 1 H), 1.44 (d, *J* = 7.1 Hz, 3 H). ^13^C NMR (125 MHz, 500 MHZ, DMSO-*d*_6_): δ = 171.6, 141.5, 140.6, 140.4, 132.9, 130.9, 129.5, 129.2, 129.0, 128.8, 128.5, 128.4, 128.3, 127.9, 126.7, 126.5, 126.4, 126.2, 126.0, 53.5, 52.1, 17.6, 14.9, 12.0. HRMS (ESI+): *m*/*z* [M + Na]^+^ calcd for C_22_H_21_NO_2_Na^+^: 354.1470; found: 354.1505.

Synthesis of (**(*R*)-*N*-hydroxy-*N*-(1-(naphthalen-1-yl)ethyl)benzamide**) (**HA4**). General procedure B afforded the product HA4 as a white solid in 88% yield. The product was purified by precipitating the product from a mixture of toluene or benzene and hexane at 0 °C.^1^H NMR (500 MHZ, DMSO-*d*_6_): δ = δ = 9.64 (br, 1H), 8.23–8.11 (m, 1H), 7.97 (dd, *J* = 7.7, 1.8 Hz, 1H), 7.90 (d, *J* = 8.2 Hz, 1H), 7.68 (d, *J* = 7.1 Hz, 1H), 7.60–7.51 (m, 5H), 7.45–7.34 (m, 3H), 6.49 (d, *J* = 7.3 Hz, 1H), 1.69 (d, *J* = 6.8 Hz, 3H). ^13^C NMR (125 MHz, DMSO-*d*_6_): δ = 168.6, 136.6, 135.5, 133.9, 131.6, 130.4, 129.2, 128.7, 128.5, 128.1, 126.8, 126.1, 125.8, 125.4, 123.7, 50.9, 17.0. HRMS (ESI+): *m*/*z* [M + Na]^+^ calcd for C_19_H_18_NO_2_Na^+^: 314.1157; found: 314.1180.

Synthesis of (**(*R*)-*N*-hydroxy-3,5-dimethyl-*N*-(1-(naphthalen-1-yl)ethyl)benzamide**) **(HA5).** General procedure B afforded the product HA5 as a white solid in 86% yield. The product was purified by precipitating the product from a mixture of toluene or benzene and hexane at 0 °C. ^1^H NMR (500 MHZ, DMSO-*d*_6_): δ = 8.50 (s, 1H), 7.92 (d, *J* = 8.2 Hz, 1H), 7.87–7.80 (m, 1H), 7.54–7.43 (m, 3H), 7.36 (s, 1H), 7.25 (dd, *J* = 5.1, 2.0 Hz, 1H), 6.93 (s, 1H), 6.66 (s, 2H), 5.65 (s, 1H), 2.03 (s, 6H), 1.66 (d, *J* = 6.7 Hz, 3H). ^13^C NMR (125 MHz, DMSO-*d*_6_): δ = 168.9, 154.8, 136.5, 133.8, 130.9, 128.9, 128.8, 126.9, 126.4, 126.4, 125.9, 125.8, 51.0, 21.2, 16.8. HRMS (ESI+): *m*/*z* [M + Na]^+^ calcd for C_21_H_22_NO_2_Na^+^: 342.1470; found: 342.1447.

Synthesis of (**(*S*)-*N*-(3,3-dimethylbutan-2-yl)-*N*-hydroxybenzamide**) (**HA6**). General procedure B afforded the product HA6 as a white solid in 98% yield. The product was purified by precipitating the product from a mixture of toluene or benzene and hexane at 0 °C. ^1^H NMR (500 MHZ, DMSO-*d*_6_): δ = 9.41 (s, 1H), 7.57–7.38 (m, 5H), 4.47 (s, 1H), 1.15 (d, *J* = 7.0 Hz, 3H), 0.96 (s, 9H). ^13^C NMR (125 MHz, DMSO-*d*_6_): δ = 169.1, 136.3, 130.0, 128.6, 128.1, 57.9, 35.4, 27.6, 12.4. HRMS (ESI+): *m*/*z* [M + Na]^+^ calcd for C_13_H_20_NO_2_Na^+^: 244.1313; found: 244.1304.

Synthesis of (**(*S*)-*N*-(3,3-dimethylbutan-2-yl)-*N*-hydroxy-3,5-dimethylbenzamide**) (**HA7**). General procedure B afforded the product HA7 as a white solid in 90% yield. The product was purified by precipitating the product from a mixture of toluene or benzene and hexane at 0 °C. ^1^H NMR (500 MHZ, DMSO-*d*_6_): δ = 9.29 (s, 1H), 7.16 (s, 2H), 7.04 (s, 1H), 4.45 (s, 1H), 2.29 (s, 6H), 1.14 (d, *J* = 6.9 Hz, 3H), 0.95 (s, 9H). ^13^C NMR (125 MHz, DMSO-*d*_6_): δ = 169.5, 137.0, 136.5, 131.1, 126.1, 57.7, 35.3, 27.6, 21.3, 12.4. HRMS (ESI+): *m*/*z* [M + Na]^+^ calcd for C_15_H_24_NO_2_Na^+^: 272.1626; found: 272.1620.

**Asymmetric Epoxidation of ((2R,3R)-3-Phenyloxiran-2-yl)methanol (9)**

To a solution of Lewis acid (0.02 mmol, 10 mol%) and HA (0.022 mmol, 11 mol%) in toluene (2 mL), (*E*)-3-phenylprop-2-en-1-ol 5a (0.2 mmol, 1 equiv) and CHP (0.24 mmol, 1.2 equiv) were added. Product **9** is obtained as a yellow oil (8.41 mg, 0.056 mmol, 28% yield). TLC was used to monitor the reaction (hexane/EtOAc 5:1) with visualization by Phosphomolybdic stain. The compound was purified by flash chromatography column (gradient hexane/EtOAc, 9:1 to 5:1). The *e.e.* was determined by SFC using a Chiralcel column [OD-H, *n*-hexane/2-propanol = 90:10]; 1 mL/min, 210 nm, retention time (2S,3S) = 12.1 min, retention time (2R,3R) = 13.49 min, *e.e.* > 71%. Characterization data are available in previous reports [17].

## 4. Conclusions

This comparative study demonstrates that ligand symmetry critically influences the stereochemical outcome of vanadium-catalyzed asymmetric epoxidations. C_2_-symmetric bishydroxamic acids (BHAs) generate rigid and well-defined chiral pockets that favor high enantioselectivity but often at the expense of catalytic turnover. Conversely, C_1_-symmetric hydroxamic acids (HAs) introduce controlled desymmetrization, affording greater substrate adaptability and, in specific cases (HA3), comparable enantiocontrol of up to 71% *e.e.* These findings align with the Sabatier principle and can be rationalized through a quadrant-based steric model, in which desymmetrization fine-tunes the accessibility and stability of the chiral environment.

Ligand desymmetrization thus emerges as a valuable strategy for balancing activity and selectivity in asymmetric catalysis. Future studies will expand the substrate scope, probe solvent–ligand–metal interactions (notably in CH_2_Cl_2_), and employ DFT calculations to validate the proposed transition-state geometries. The present work was performed under unified catalytic conditions to ensure direct comparison between ligand classes. Ongoing optimization of the C_2_-symmetric BHA system (varying solvent, temperature, oxidant activation, and precomplexation) will provide a rigorous benchmark for assessing the full potential of both ligand families in asymmetric oxidation chemistry.

## Figures and Tables

**Table 1:** Comparison of enantioselectivities using BHA1, BHA2 and HA3.

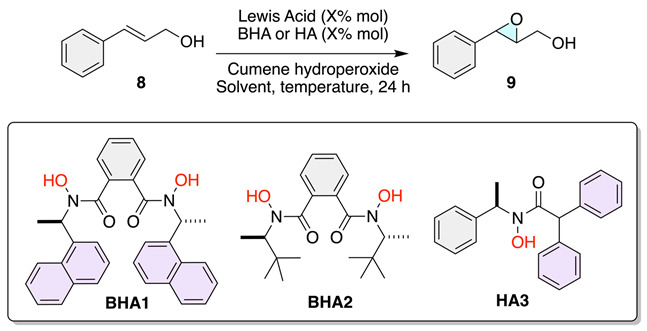						
Entry	Lewis Acid (% mol)	HA (% mol)	Solvent	Temperature (°C)	Conversion (%)	*e.e.* ^a^ (%)
1	VO(O*i*Pr)_3_ (10)	BHA1 (10)	CH_2_Cl_2_	−20	14	9
2	VO(O*i*Pr)_3_ (10)	BHA2 (10)	CH_2_Cl_2_	−20	11	7
3	VO(O*i*Pr)_3_ (10)	HA3 (12)	CH_2_Cl_2_	−20	n.r.	n.d. ^b^
4	VO(O*i*Pr)_3_ (10)	HA3 (12)	CH_2_Cl_2_	r.t.	≥99	71 ^c^

^a^ The enantiomeric excesses (*e.e.*) were determined by chiral HPLC. ^b^ Not determined. ^c^ The absolute configuration of the epoxide is (*R*,*R*).

**Table 2:** Analysis of chiral hydroxamic acid architecture and Lewis Acids.

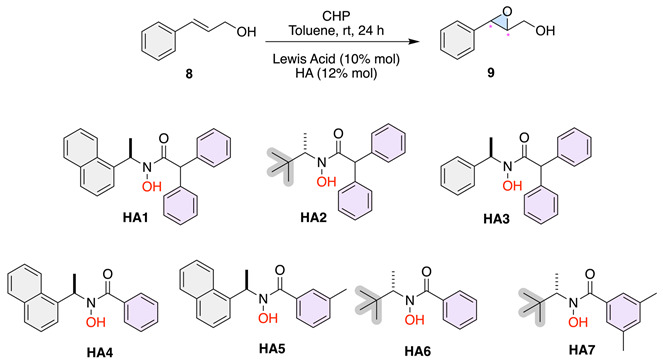				
Entry	Lewis Acid (10% mol)	HA (12% mol)	Conversion	*e.e.* ^a^
1	VO(O*i*Pr)_3_	HA1	≥99	15
2	Ti(O*i*Pr)_4_	HA2	0	n.d.
3	VO(O*i*Pr)_3_	HA2	≥99	22
4	VO(acac)_2_	HA2	≥99	19
5	Ti(O*i*Pr)_4_	HA3	0	n.d. ^b^
6	VO(O*i*Pr)_3_	HA3	≥99	19
7	VO(O*i*Pr)_3_	HA4	≥99	11
8	VO(O*i*Pr)_3_	HA5	≥99	30
9	VO(O*i*Pr)_3_	HA6	≥99	4
10	VO(O*i*Pr)_3_	HA7	≥99	49

^a^ The enantiomeric excesses (*e.e.*) were determined by chiral HPLC. ^b^ Not determined.

**Table 3:** Analysis of solvent, additive, and temperature effects in the epoxidation reaction.

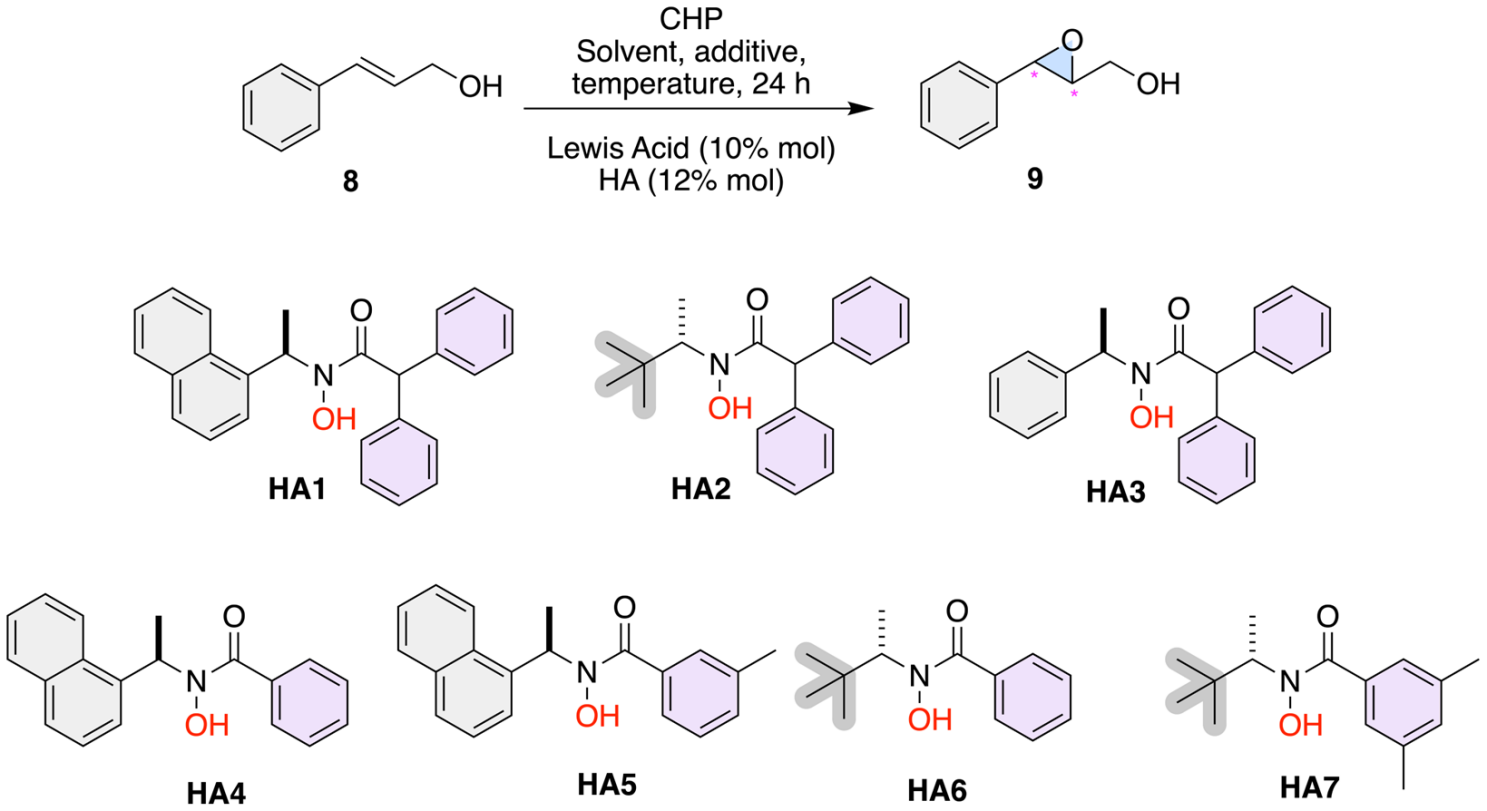							
Entry	Lewis Acid (% mol)	HA (% mol)	Solvent	Additive	Temperature	Conversion (%)	*e.e.* ^a^ (%)
**1**	VO(O*i*Pr)_3_ (10)	HA2 (12)	CH_2_Cl_2_	-	r.t.	≥99	23
**2**	VO(O*i*Pr)_3_ (10)	HA2 (12)	CH_3_CN	-	r.t.	≥99	4
**3**	VO(O*i*Pr)_3_ (10)	HA3 (12)	CH_2_Cl_2_	-	r.t.	≥99	71 ^b^
**4**	VO(O*i*Pr)_3_ (10)	HA3 (12)	CH_3_CN	-	r.t.	≥99	63
**5**	VO(O*i*Pr)_3_ (5)	HA2 (7)	Toluene	-	r.t.	≥99	8
**6**	VO(O*i*Pr)_3_ (5)	HA2 (7)	Toluene	-	r.t.	≥99	55 ^c^
**7**	Ti(O*i*Pr)_4_ (1.05 eq)	HA3 (1.05 eq)	CH_2_Cl_2_	-	r.t.	0	n.d. ^d^
**8**	VO(O*i*Pr)_3_ (10)	HA2 (12)	Toluene	MS 4Å	r.t.	≥99	18
**9**	VO(O*i*Pr)_3_ (10)	HA2 (12)	Toluene	MgO	r.t.	≥99	22
**10**	VO(O*i*Pr)_3_ (10)	HA2 (20)	Toluene	-	r.t.	≥99	25
**11^a^**	VO(O*i*Pr)_3_ (10)	HA7 (20)	Toluene	-	r.t.	≥99	7
**12**	VO(O*i*Pr)_3_ (10)	HA1 (12)	Toluene	-	0 °C	≥99	19
**13**	VO(O*i*Pr)_3_ (10)	HA3 (12)	Toluene	-	0 °C	≥99	23
**14**	VO(O*i*Pr)_3_ (10)	HA2 (12)	Toluene	-	0 °C	≥99	21
**15**	VO(O*i*Pr)_3_ (10)	HA6 (12)	Toluene	-	0 °C	≥99	8
**16**	VO(O*i*Pr)_3_ (10)	HA7 (12)	Toluene	-	0 °C	≥99	4
**17**	VO(O*i*Pr)_3_ (10)	HA4 (12)	Toluene	-	0 °C	≥99	14
**18**	VO(O*i*Pr)_3_ (10)	HA5 (12)	Toluene	-	0 °C	≥99	24
**19**	VO(acac)_2_ (10)	HA2 (12)	Toluene	-	0 °C	≥99	17

^a^ The enantiomeric excesses (*e.e.*) were determined by chiral HPLC. ^b^ The absolute configuration of the epoxide is (*R*,*R*). ^c^ The reaction time was 48 h. ^d^ Not determined.

## Data Availability

The original contributions presented in this study are included in the article. Further inquiries can be directed to the corresponding author.

## Supplementary Materials

The following supporting information can be downloaded at: https://www.mdpi.com/article/10.3390/molecules30214311/s1.
