# Supplementary material for: A Comparative Study of C2-Symmetric and C1-Symmetric Hydroxamic Acids in Vanadium-Catalyzed Asymmetric Epoxidation of Allylic Alcohols
Source: Molecules. 2025 Nov 6;30(21):4311. doi: 10.3390/molecules30214311 (PMC12609139; doi:10.3390/molecules30214311)

A Comparative Study of C_2-symmetric and C_1-symmetric Hydroxamic Acids in Vanadium-Catalyzed Asymmetric Epoxidation of Allylic Alcohols

Valtierra-Galvan, M. F.; [1] Rodriguez-Hernandez, A.; [1] Bonilla-Landa, I.; [1] Barrera-Mendez, F.; [1,4] Enríquez-Medrano, F. J.; [2] Díaz de Leon-Gomez, R. E.; [2] Olivares- Romero and J.L. [1,*]

[1]Red de Estudios Moleculares Avanzados, Campus III. Instituto de Ecología, A. C., Carretera Antigua a Coatepec 351, 91073, Xalapa, Veracruz, México.
[2]Research Center in Applied Chemistry (CIQA), Enrique Reyna Hermosillo, No. 140. Col. San José de los Cerritos, Saltillo, 25294, México
[3]Investigador CONAHCyT por México
*Correspondence: author (jose.olivares@inecol.mx)

Supporting Information

1. Representative HPLC Chromatograms

Chromatogram HPLC of 9 racemic CHIRALCEL OD-H, Hex/IPA = 90/10, 1 mL/min, 210 nm

<Chromatogram>

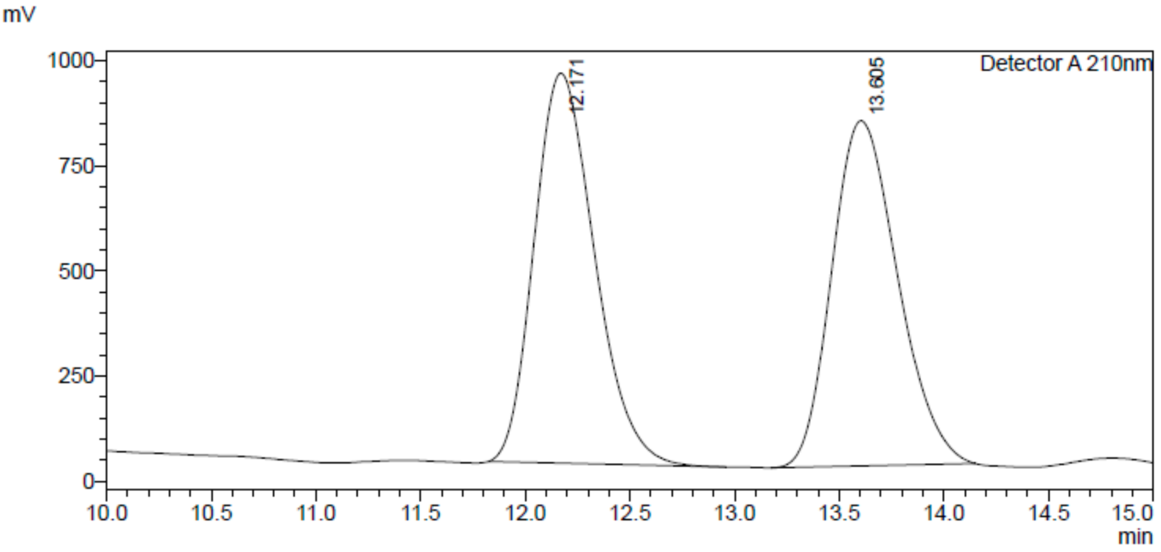

<Peak Table>

Detector A 210nm

Peak#	Ret. Time	Area	Height	Conc.	Unit	Mark	Name
1	12.171	18661496	927515	50.906		M	
2	13.605	17997342	821301	49.094		M	
Total		36658838	1748816	*e.e.*= 0%= racemic			

Chromatogram HPLC of 9 enantiomeric CHIRALCEL OD-H, Hex/IPA = 90/10, 1 mL/min, 210 nm

<Chromatogram>

10% mol $VO(OiPr)_3$
12% mol HA3
In CH_2Cl_2 at r.t.

mV

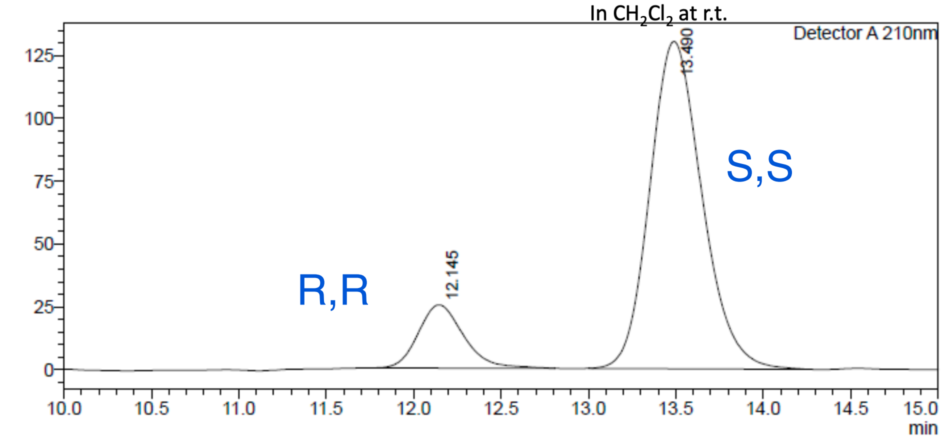

<Peak Table>

Detector A 210nm

Peak#	Ret. Time	Area	Height	Conc.	Unit	Mark	Name
1	12.145	453176	25129	14.335		M	
2	13.490	2708160	130212	85.665		M	
Total		3161335	155341	*e.e.*= 71%			

Chromatogram HPLC of 9 enantiomeric CHIRALCEL OD-H, Hex/IPA = 90/10, 1 mL/min, 210 nm

<Chromatogram>

10% mol $VO(OiPr)_3$
12% mol HA2

mV

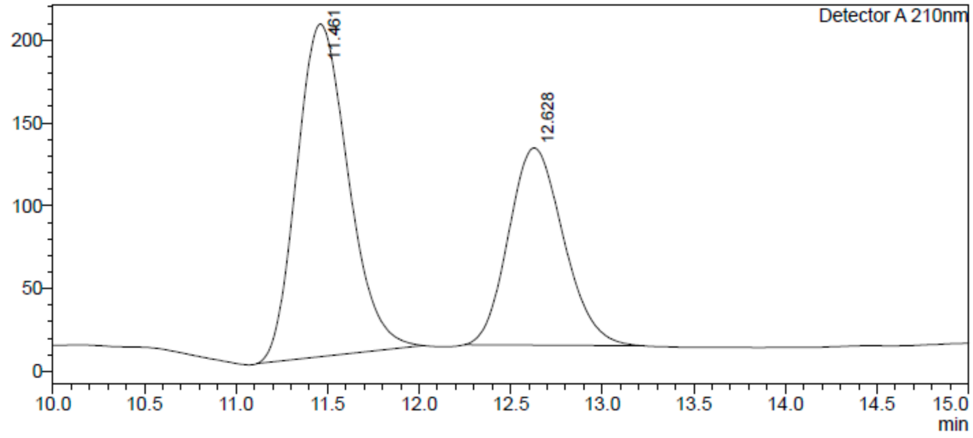

<Peak Table>

Detector A 210nm

Peak#	Ret. Time	Area	Height	Conc.	Unit	Mark	Name
1	11.461	3924456	200866	61.220		M	
2	12.628	2485994	119039	38.780		M	
Total		6410450	319906	*e.e.*= 22%			

Chromatogram HPLC of 9 enantiomeric CHIRALCEL OD-H, Hex/IPA = 90/10, 1 mL/min, 210 nm

<Chromatogram>

10% mol $VO(acac)_2$
12% mol HA2

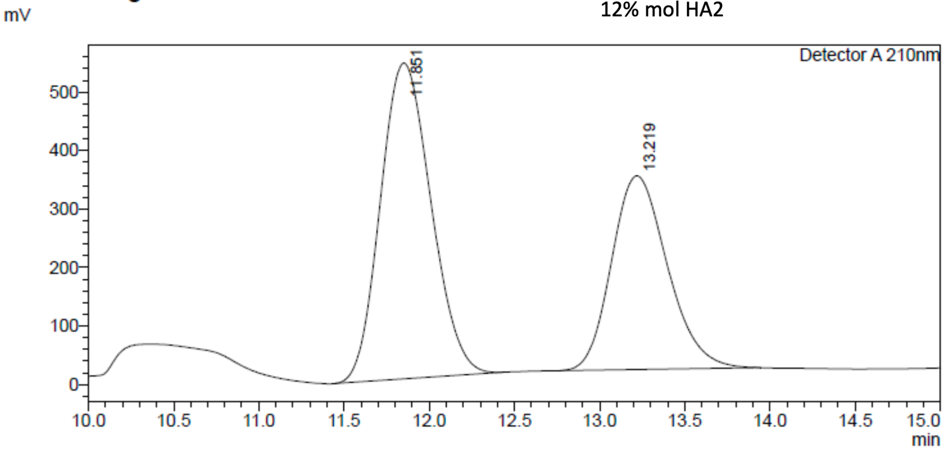

<Peak Table>

Detector A 210nm

Peak#	Ret. Time	Area	Height	Conc.	Unit	Mark	Name
1	11.851	11157872	539761	59.688		M	
2	13.219	7535745	330939	40.312		M	
Total		18693617	870700	*e.e.*= 19%			

Chromatogram HPLC of 9 enantiomeric CHIRALCEL OD-H, Hex/IPA = 90/10, 1 mL/min, 210 nm

<Chromatogram>

10% mol $VO(O\textit{i}Pr)_3$
12% mol HA2
In toluene with MgO at r.t.

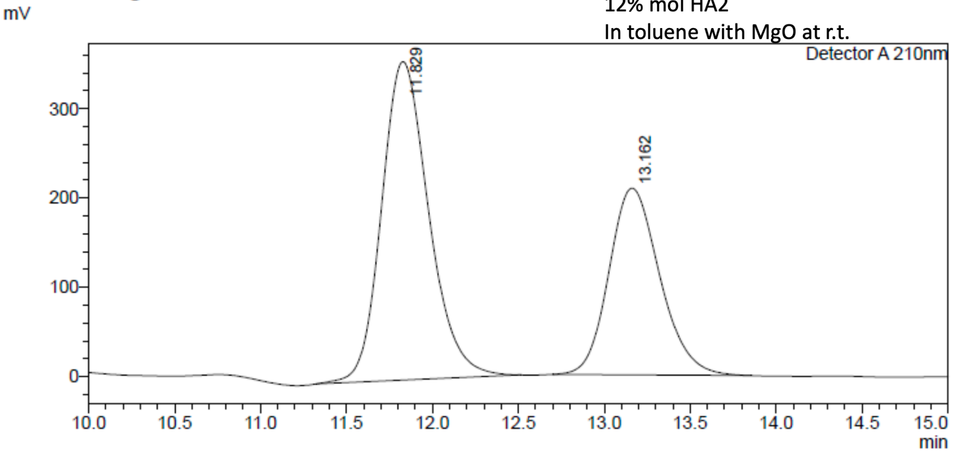

<Peak Table>

Detector A 210nm

Peak#	Ret. Time	Area	Height	Conc.	Unit	Mark	Name
1	11.829	6812377	356575	61.273		M	
2	13.162	4305695	209123	38.727		M	
Total		11118073	565699	*e.e.*= 22%			

Chromatogram HPLC of 9 enantiomeric CHIRALCEL OD-H, Hex/IPA = 90/10, 1 mL/min, 210 nm

10% mol $VO(O\textit{i}Pr)_3$
20% mol HA2
In toluene at r.t.

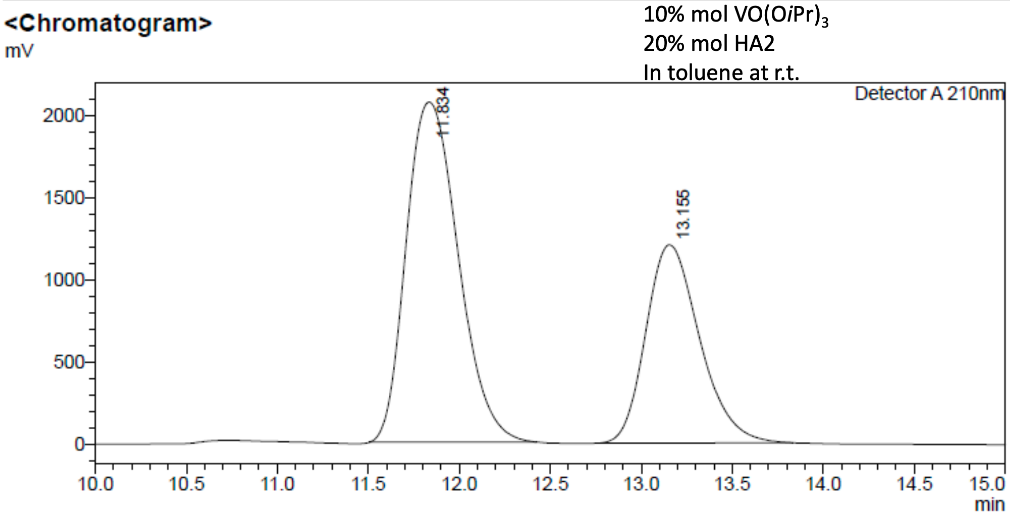

<Peak Table>

Detector A 210nm

Peak#	Ret. Time	Area	Height	Conc.	Unit	Mark	Name
1	11.834	41290648	2068263	62.470		M	
2	13.155	24806559	1205512	37.530		M	
Total		66097207	3273775	*e.e.*= 25%			

Chromatogram HPLC of 9 enantiomeric CHIRALCEL OD-H, Hex/IPA = 90/10, 1 mL/min, 210 nm

10% mol $VO(O\textit{i}Pr)_3$
12% mol HA5
In toluene at 0 °C

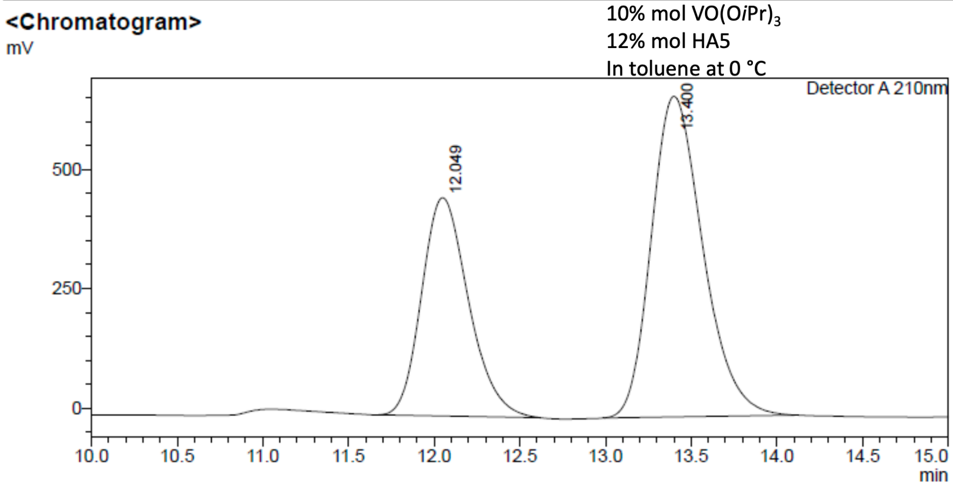

<Peak Table>

Detector A 210nm

Peak#	Ret. Time	Area	Height	Conc.	Unit	Mark	Name
1	12.049	8706211	456794	38.214		M	
2	13.400	14076538	671330	61.786		M	
Total		22782749	1128124	*e.e.*= 23%			

^{1}H NMR Spectrum (500 MHz, $CDCl_3$, 25 °C) of 1a

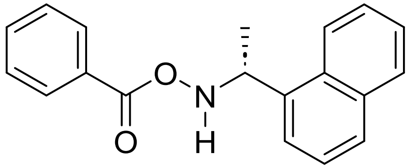

1a

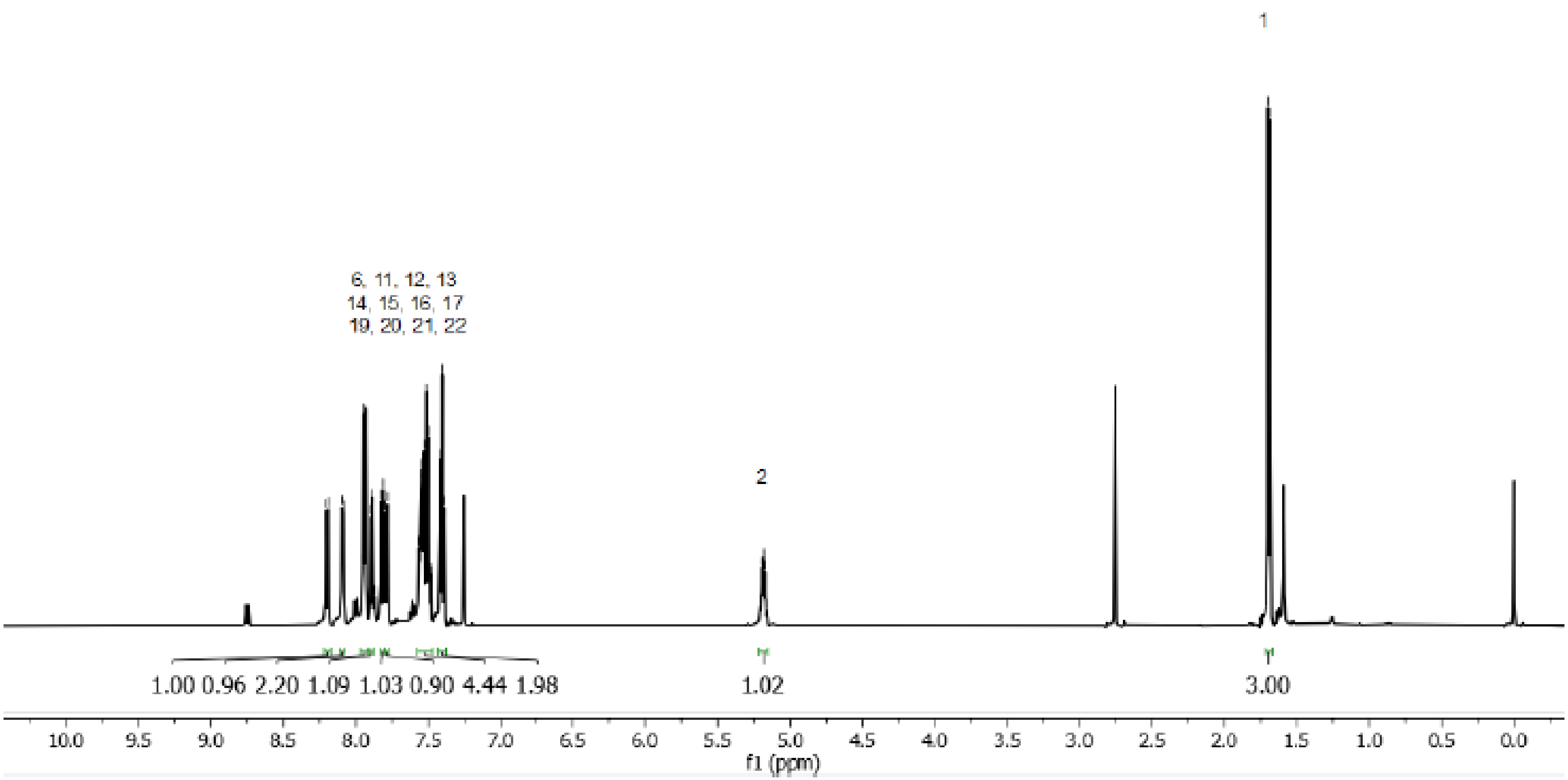

DEPTQ135 NMR Spectrum (500 MHz, $CDCl_3$, 25 °C) of 1a

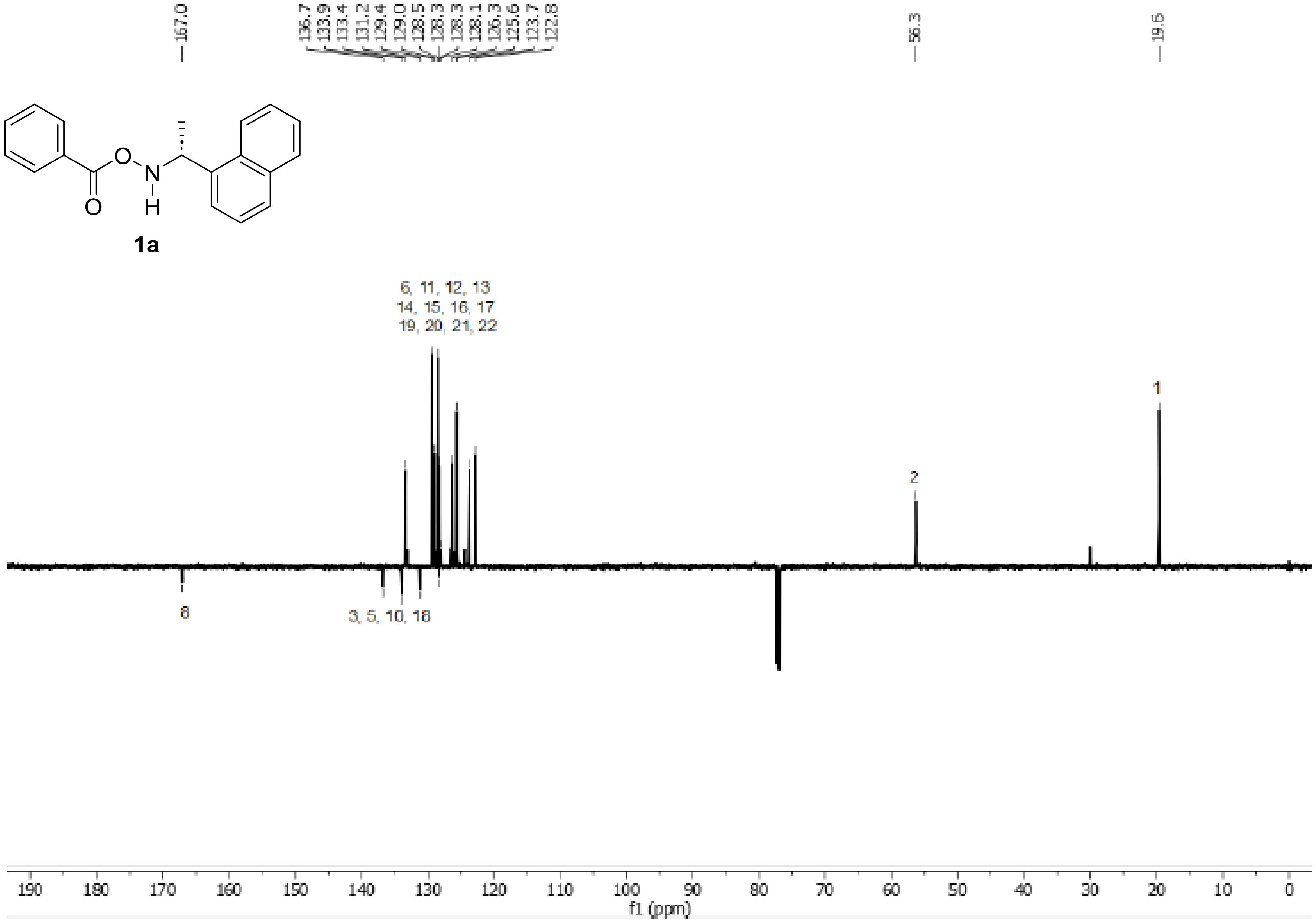

^{1}H NMR Spectrum (500 MHz, $CDCl_3$, 25 °C) of 2a

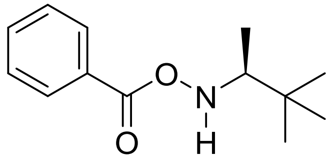

2a

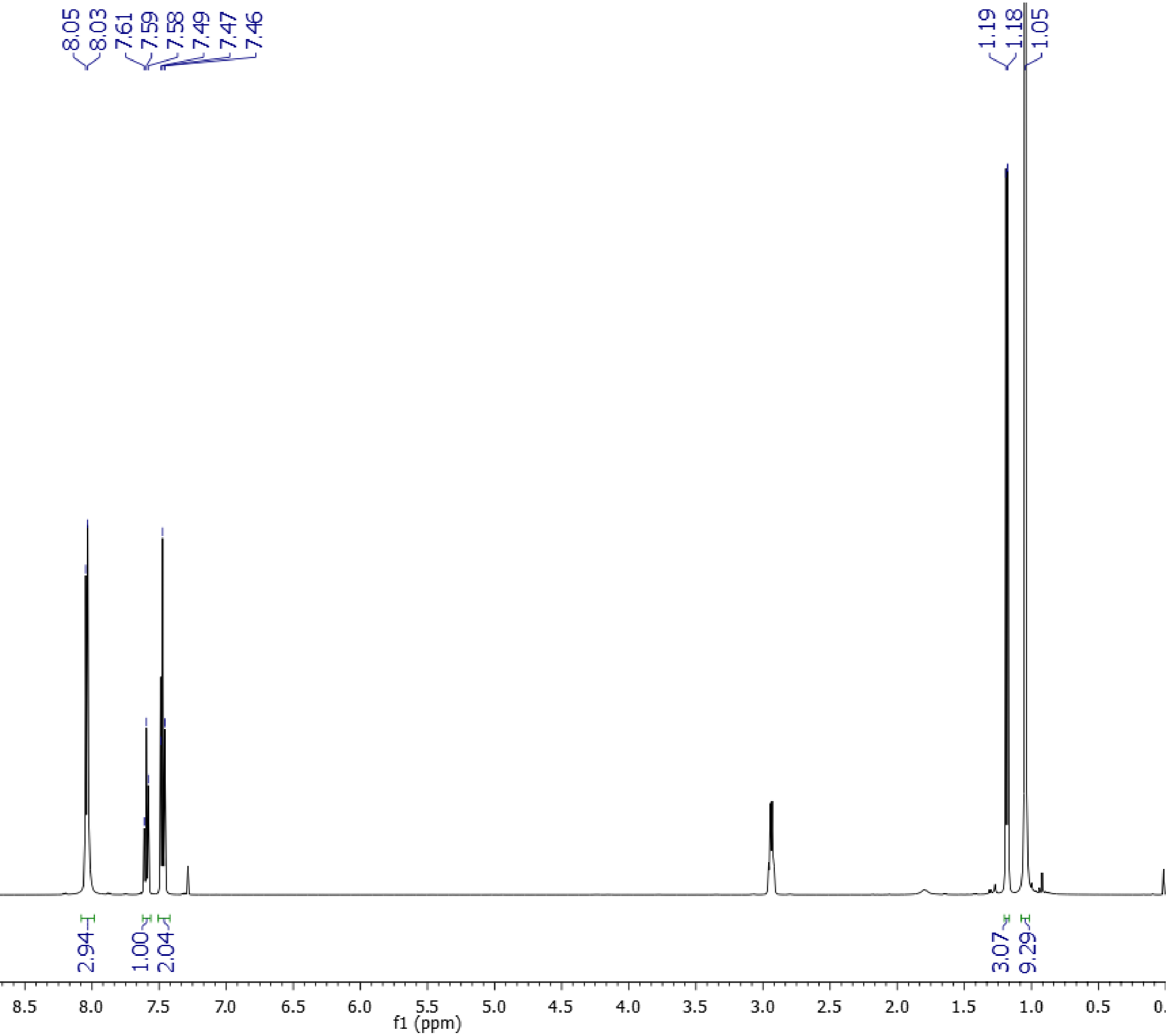

DEPTQ135 NMR Spectrum (500 MHz, $CDCl_3$, 25 °C) of 2a

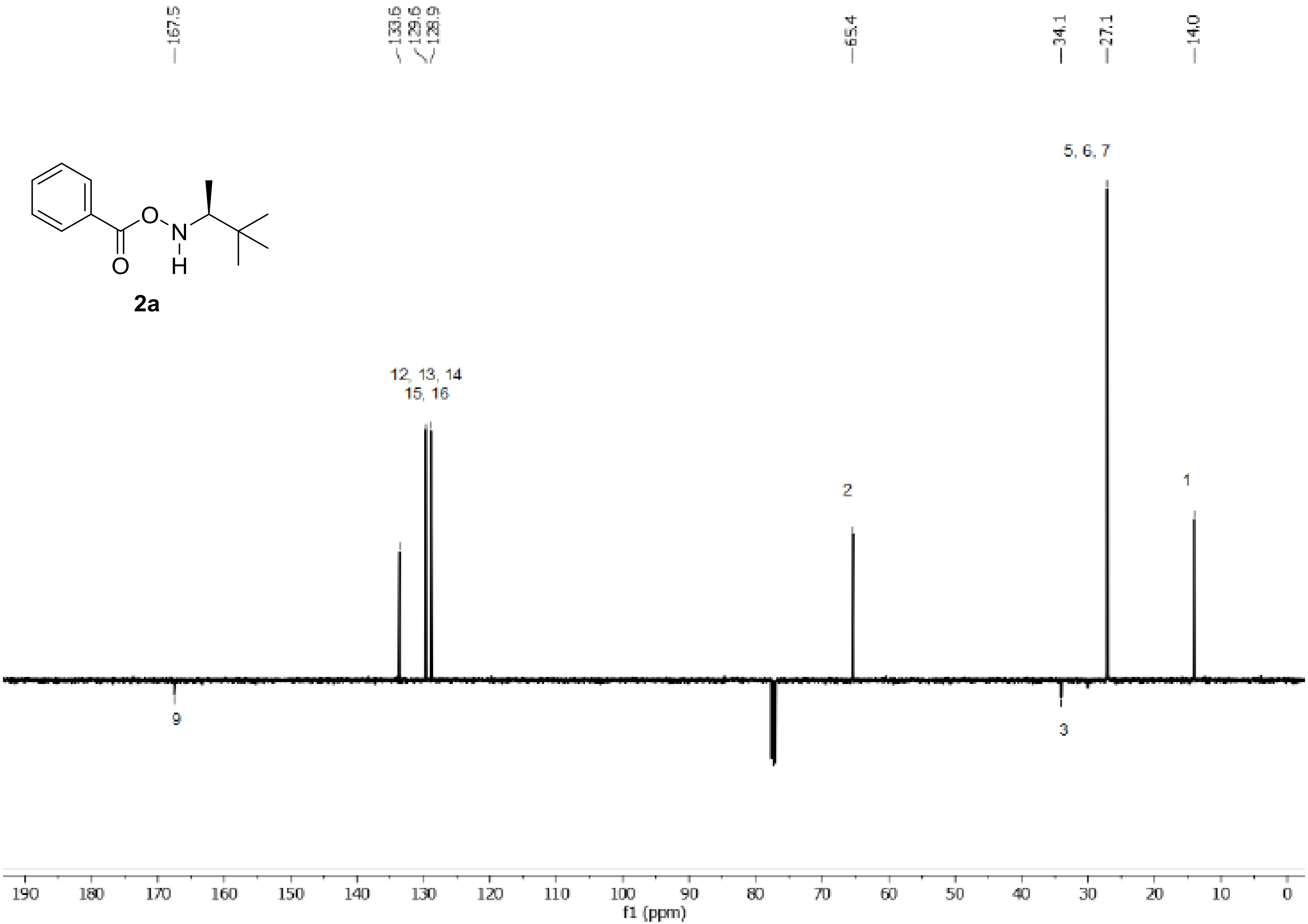

^1H NMR Spectrum (500 MHz, $CDCl_3$, 25 °C) of 3a

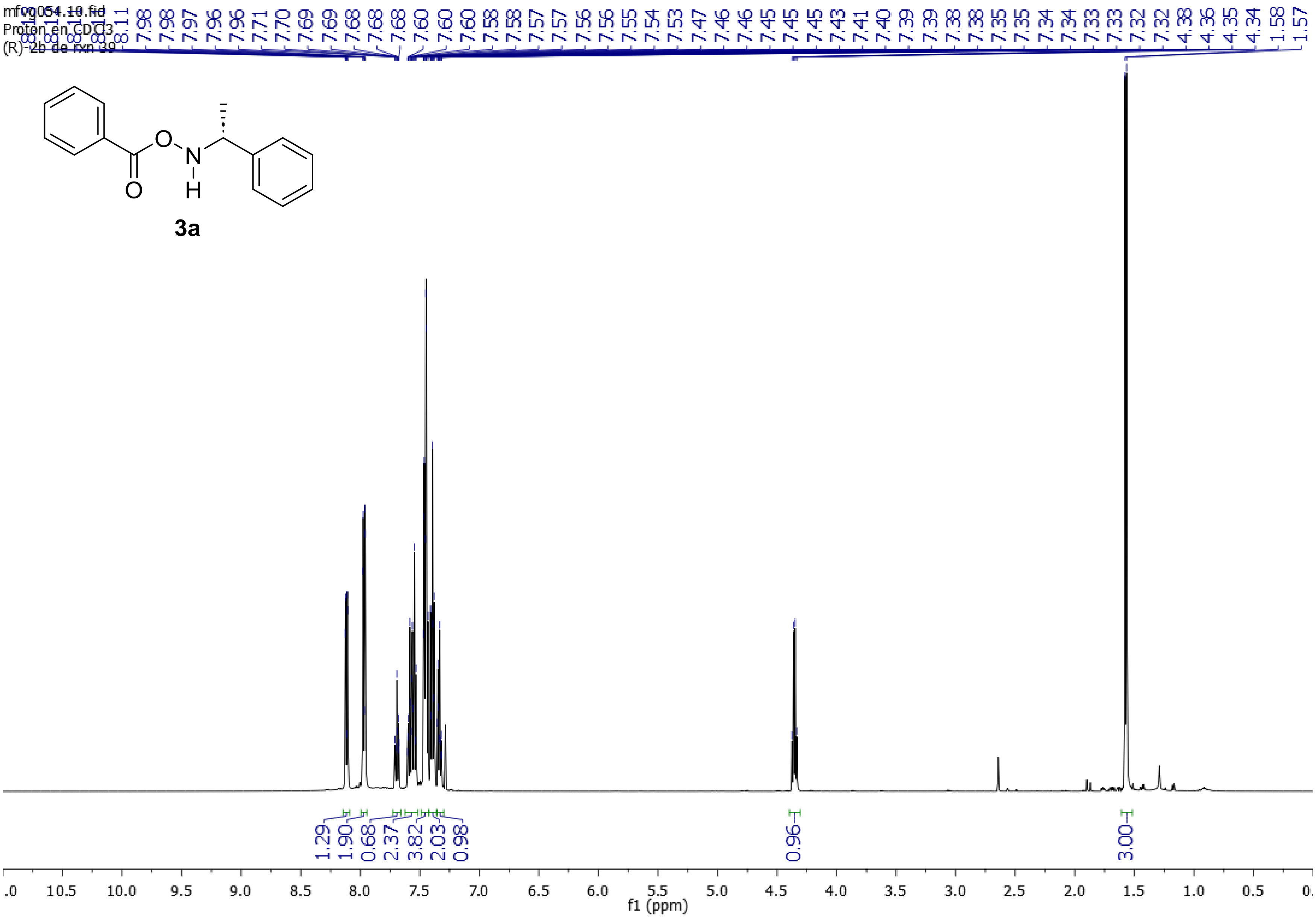

^{1}H NMR Spectrum (500 MHz, DMSO-d_6, 25 °C) of HA1

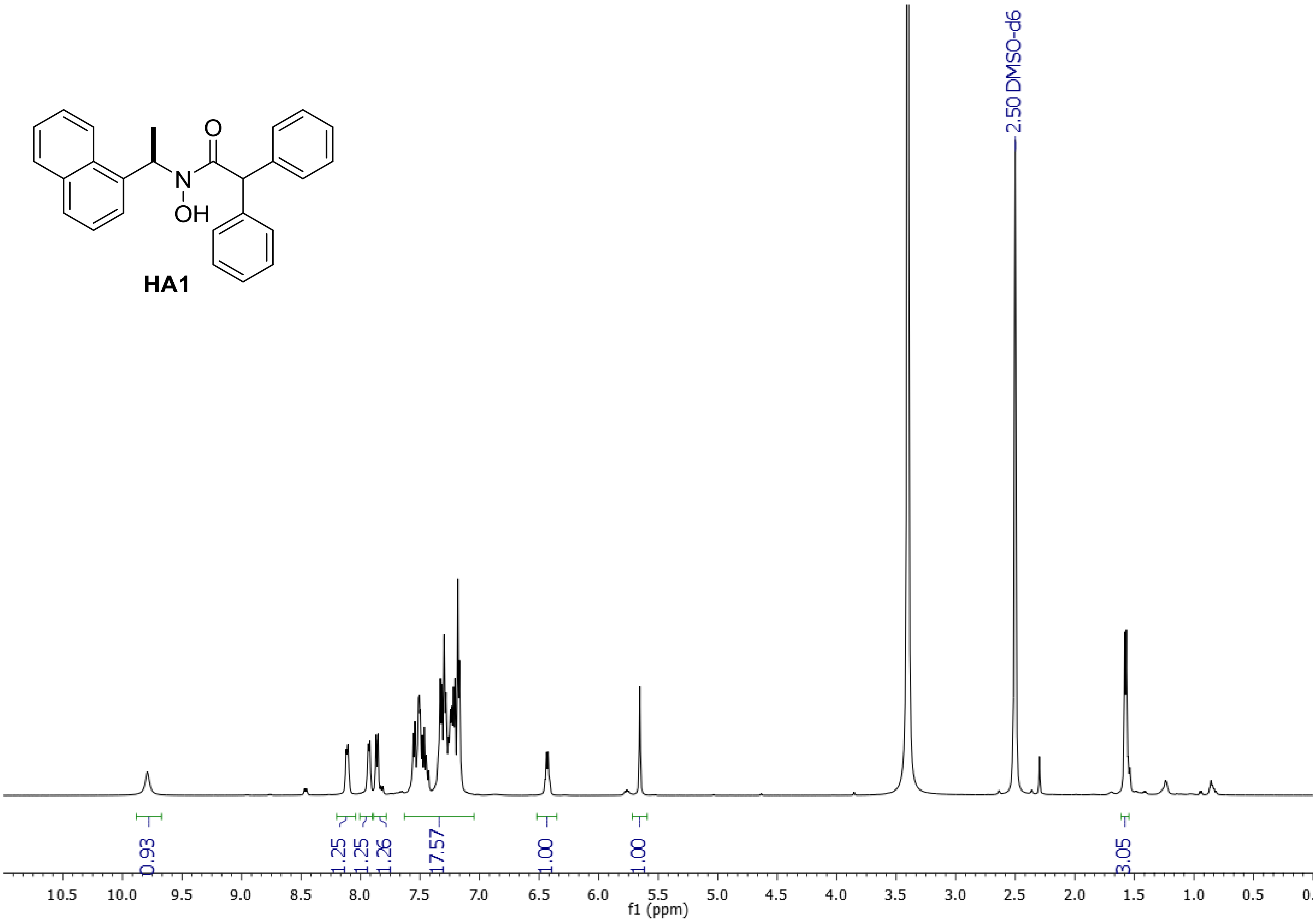

DEPTQ135 NMR Spectrum (500 MHz, DMSO-d_6, 25 °C) of HA1

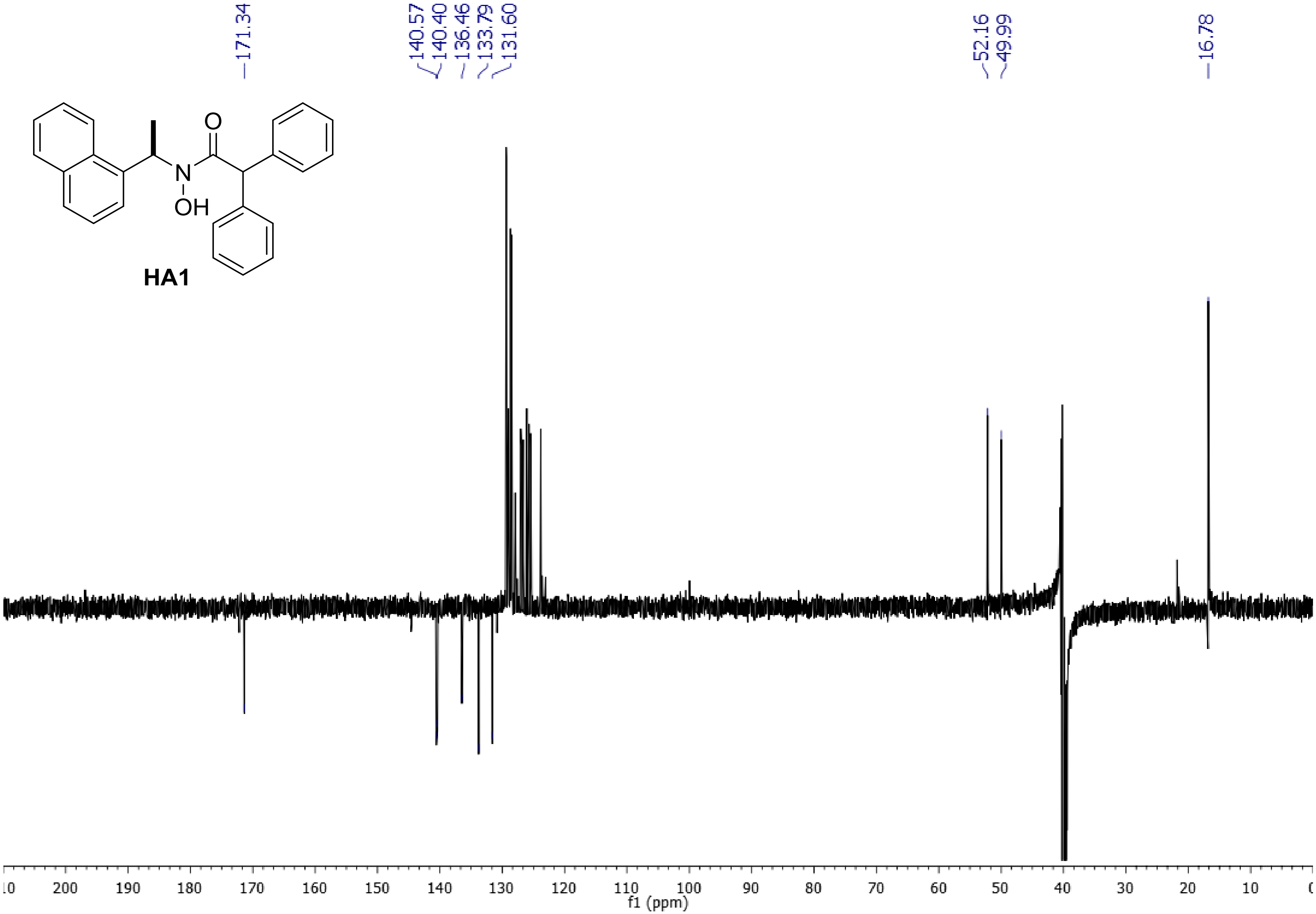

^{1}H NMR Spectrum (500 MHz, DMSO-d_6, 25 °C) of HA2

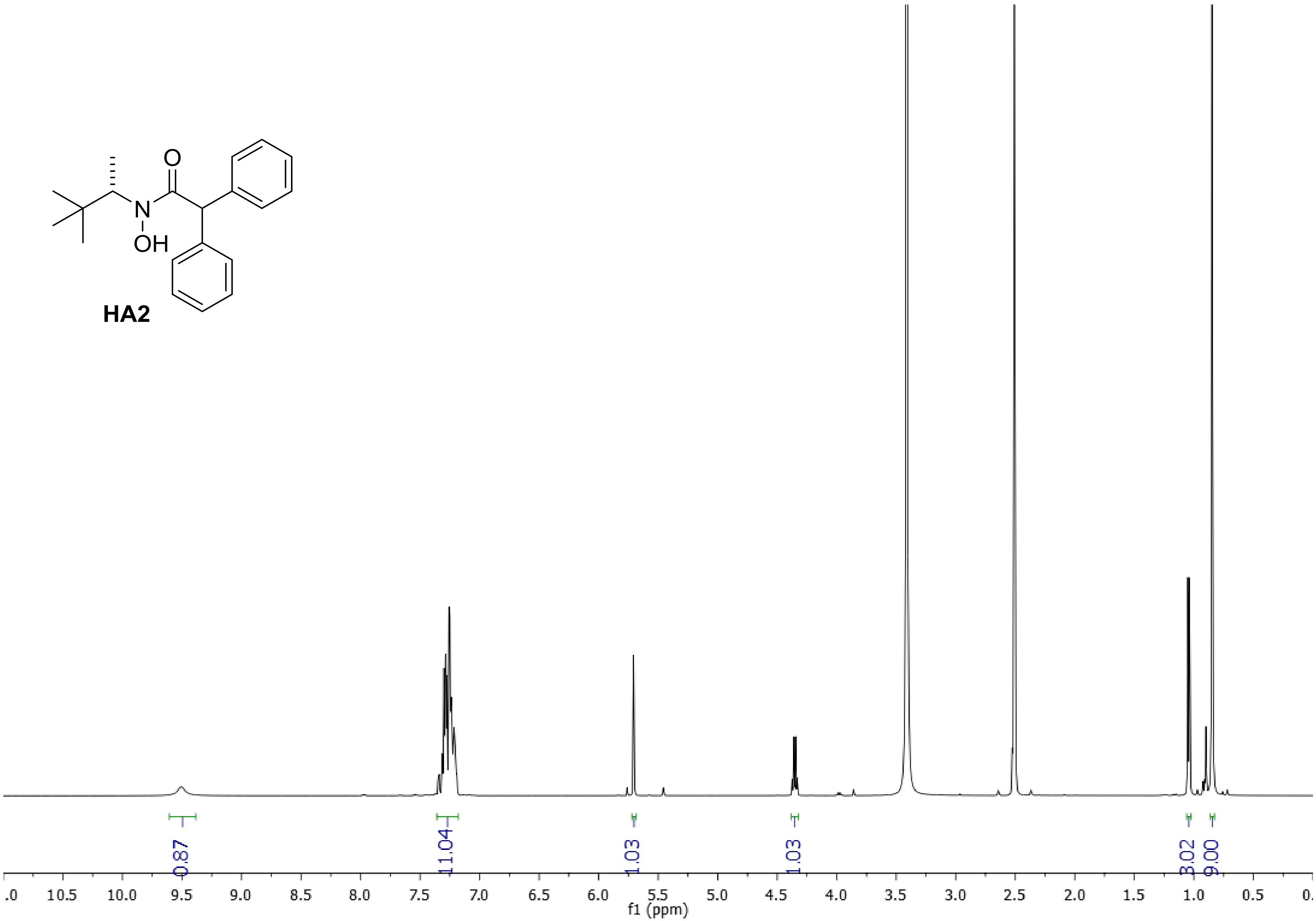

DEPTQ135 NMR Spectrum (500 MHz, DMSO-d_6, 25 °C) of HA2

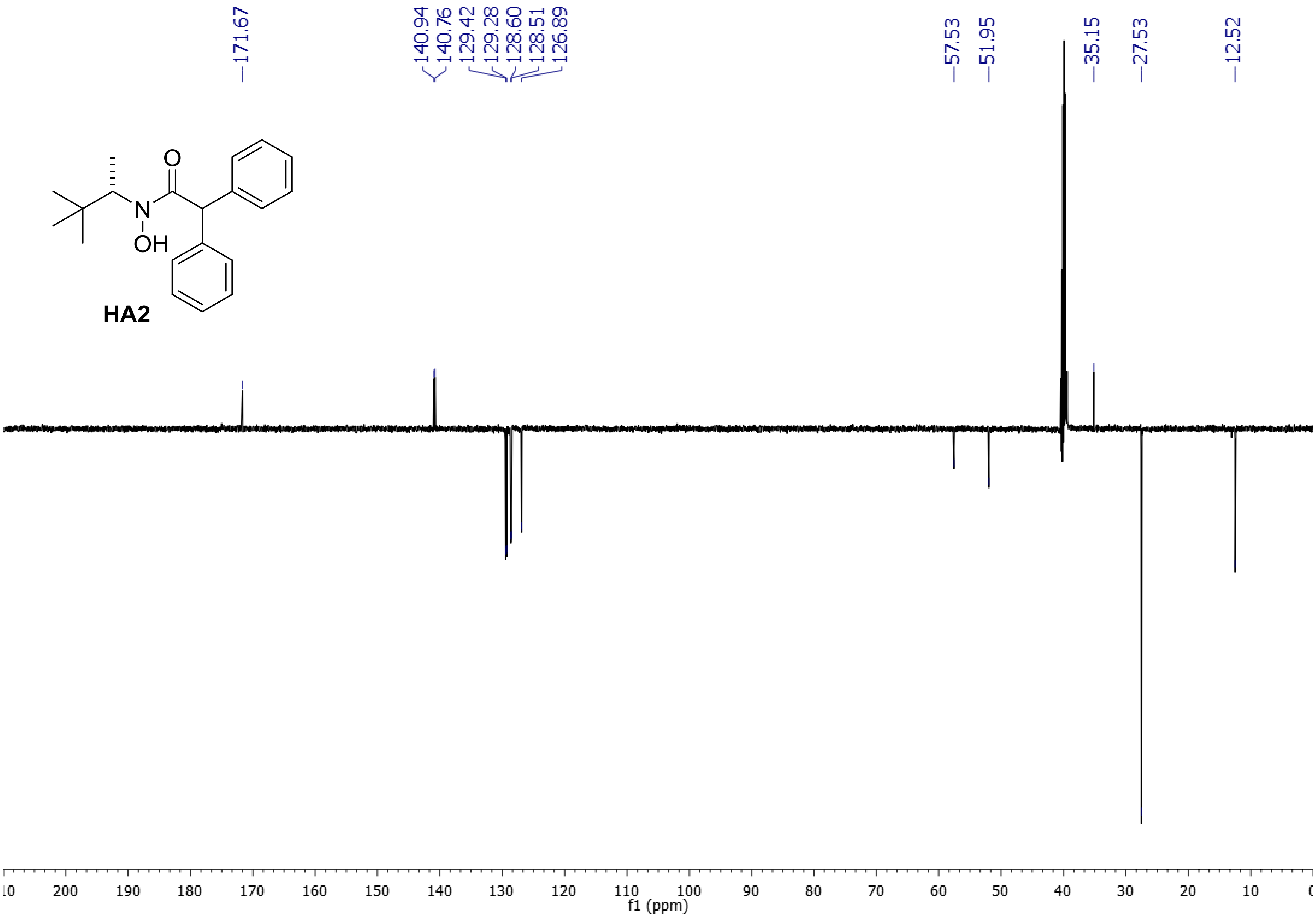

^{1}H NMR Spectrum (500 MHz, $CDCl_3$, 25 °C) of HA3

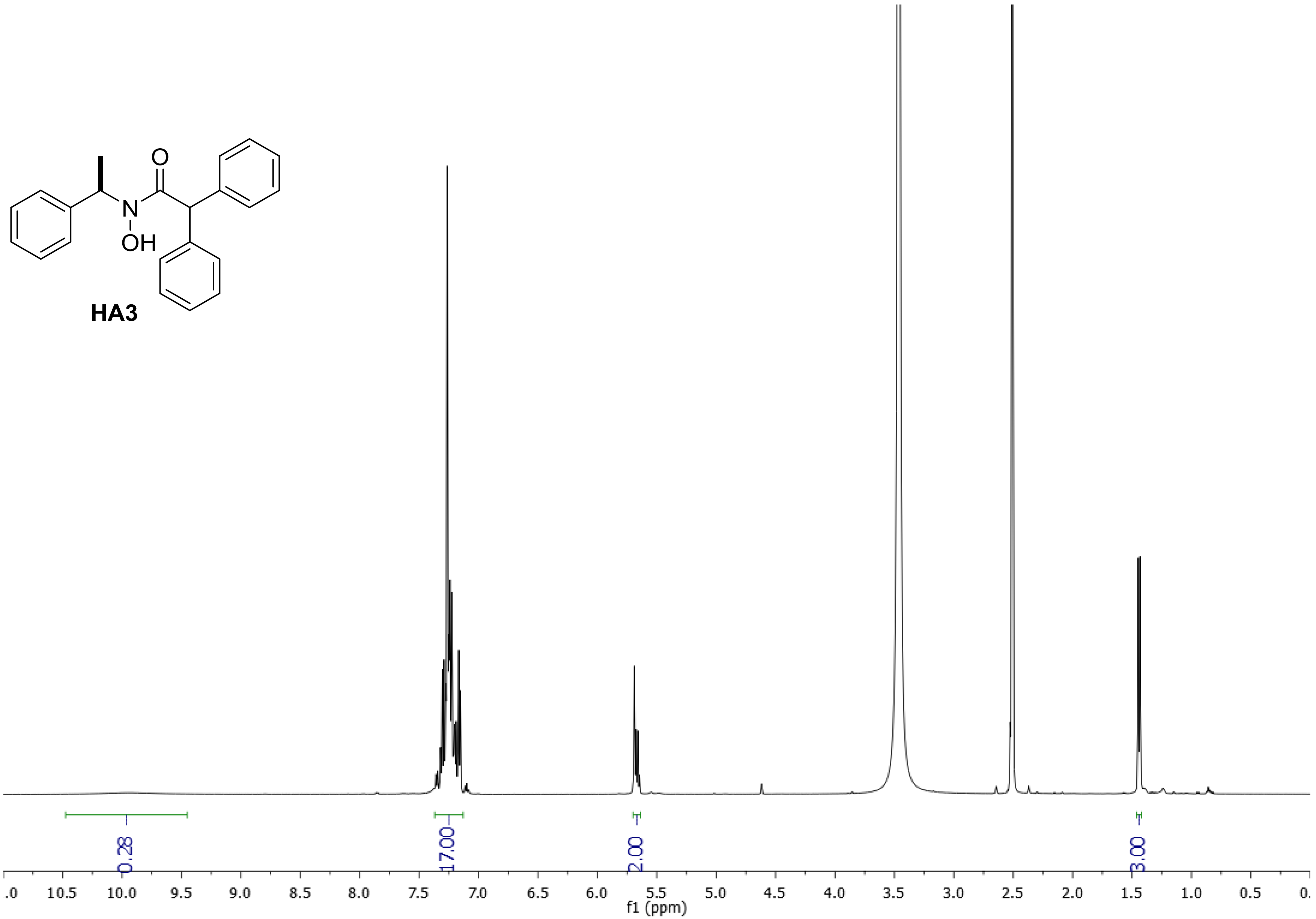

DEPTQ135 NMR Spectrum (500 MHz, $CDCl_3$, 25 °C) of HA3

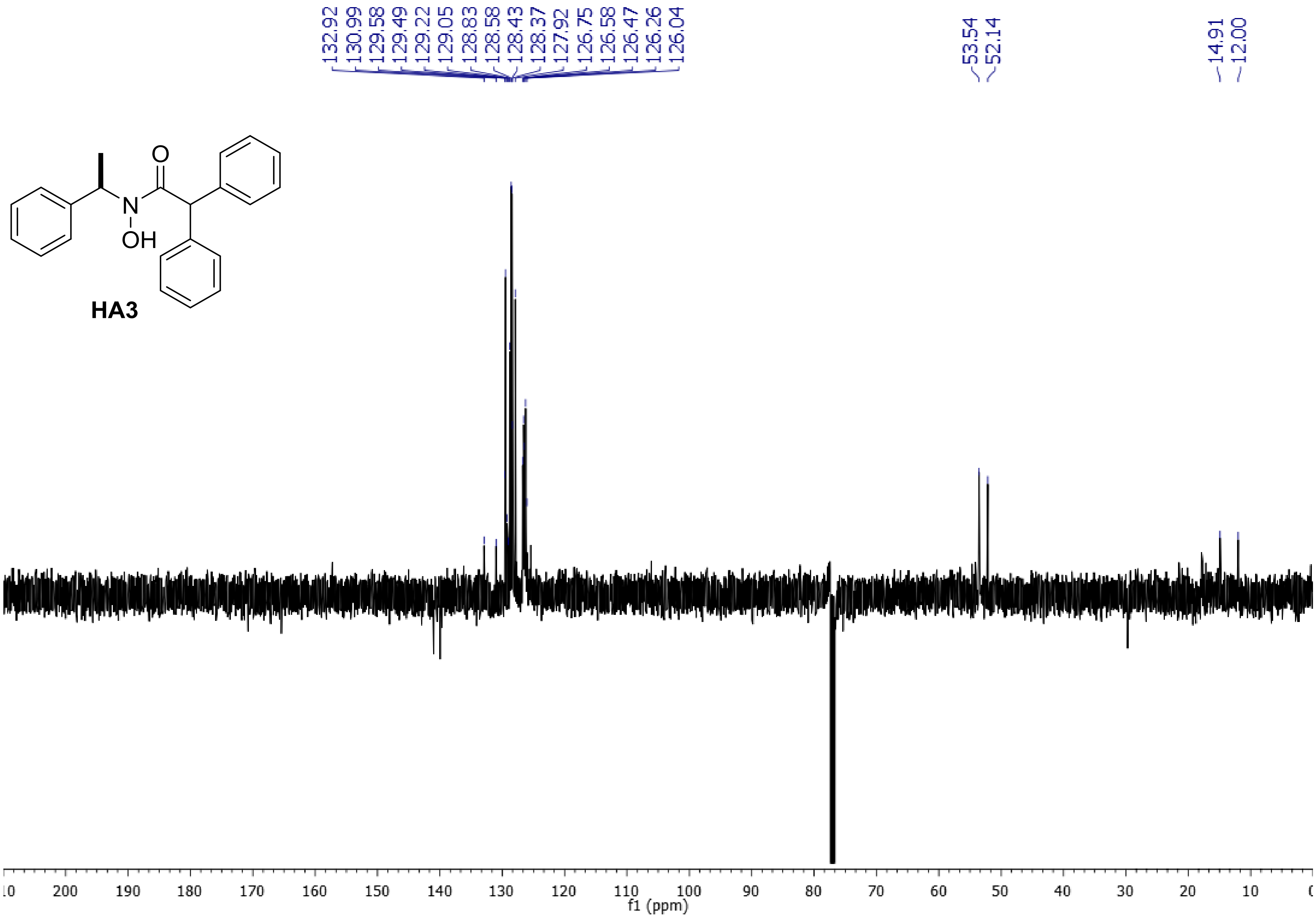

^{1}H NMR Spectrum (500 MHz, DMSO-d_6, 25 °C) of HA4

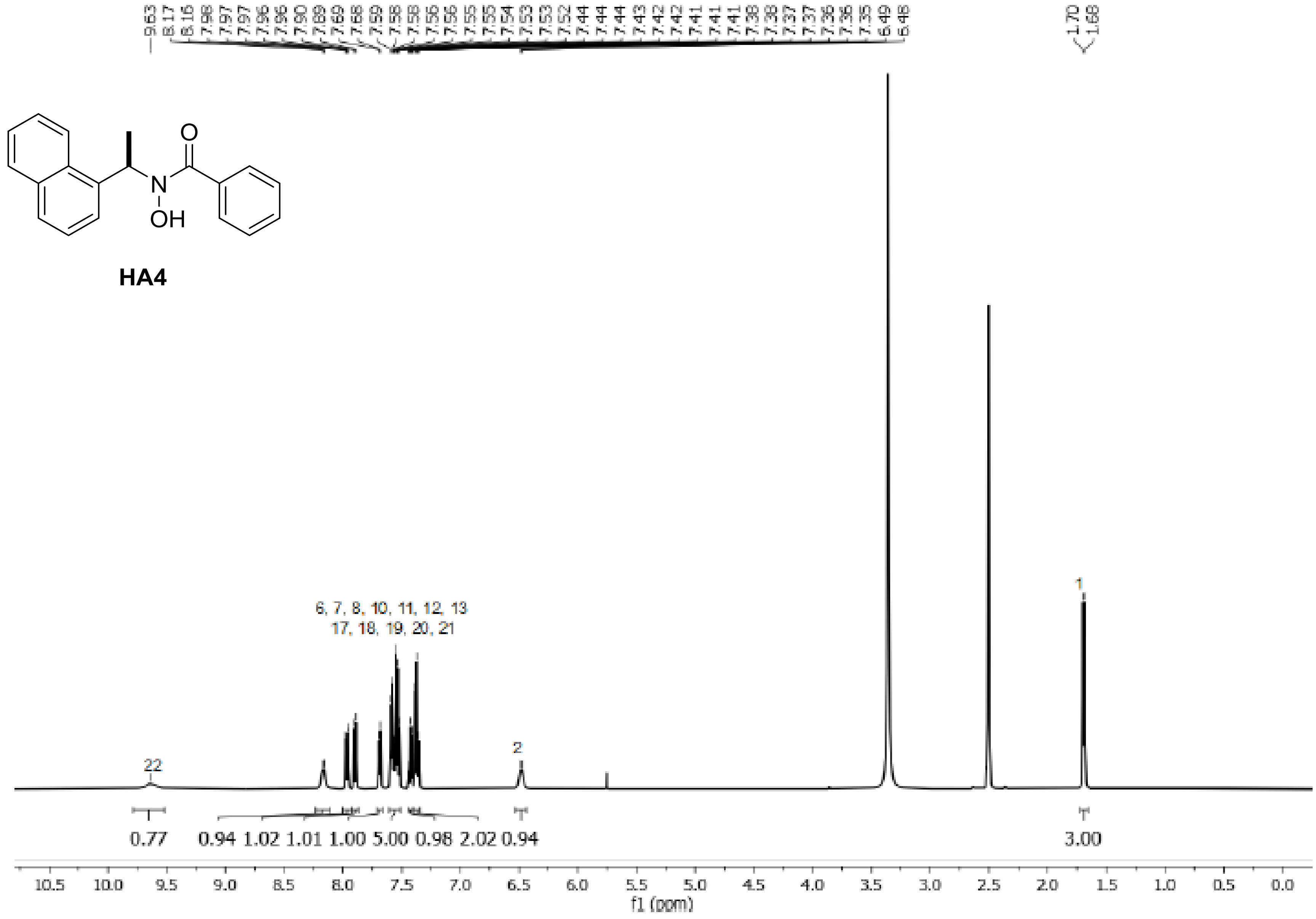

DEPTQ135 NMR Spectrum (500 MHz, DMSO-d_6, 25 °C) of HA4

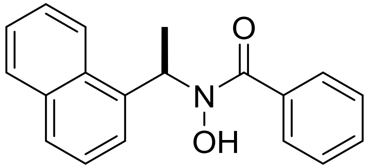

HA4

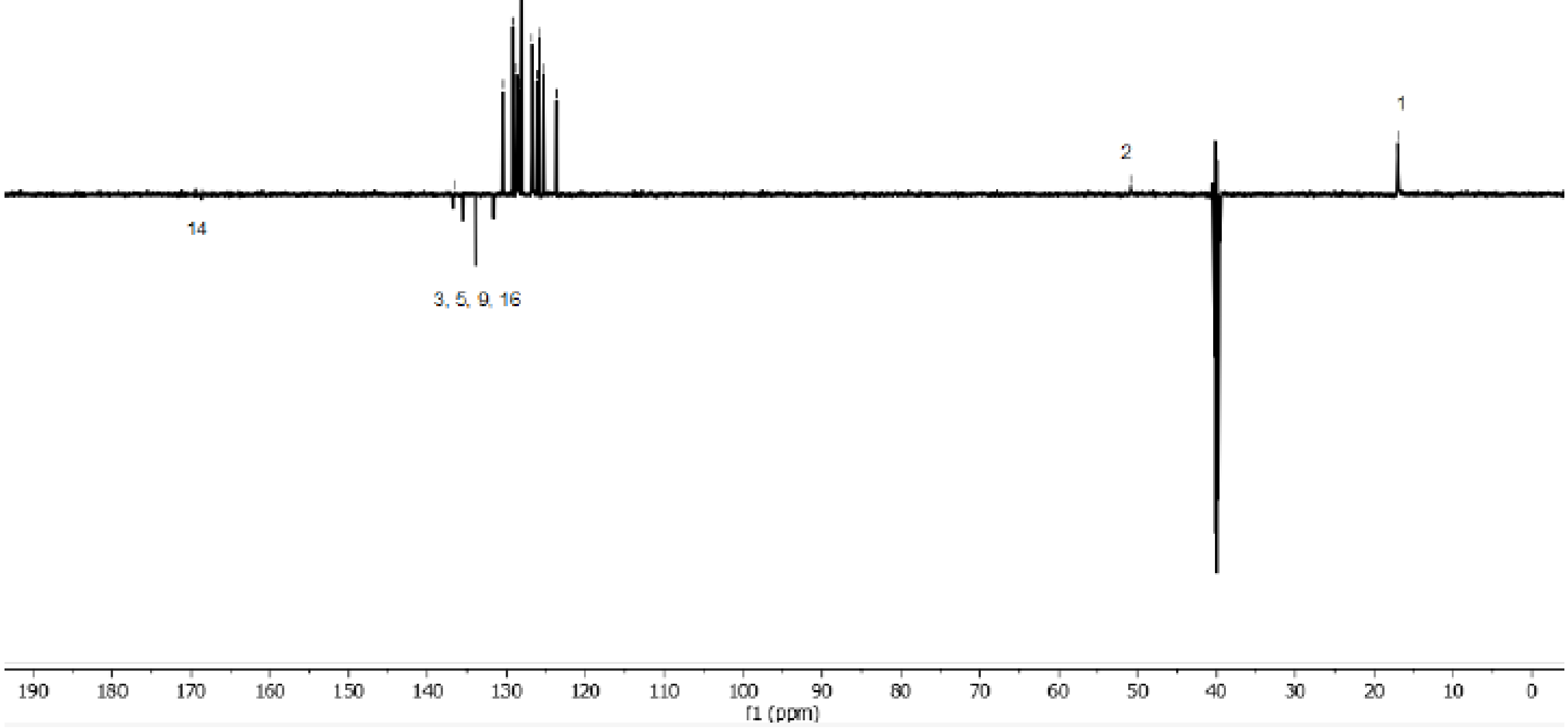

^{1}H NMR Spectrum (500 MHz, DMSO-d_6, 25 °C) of HA5

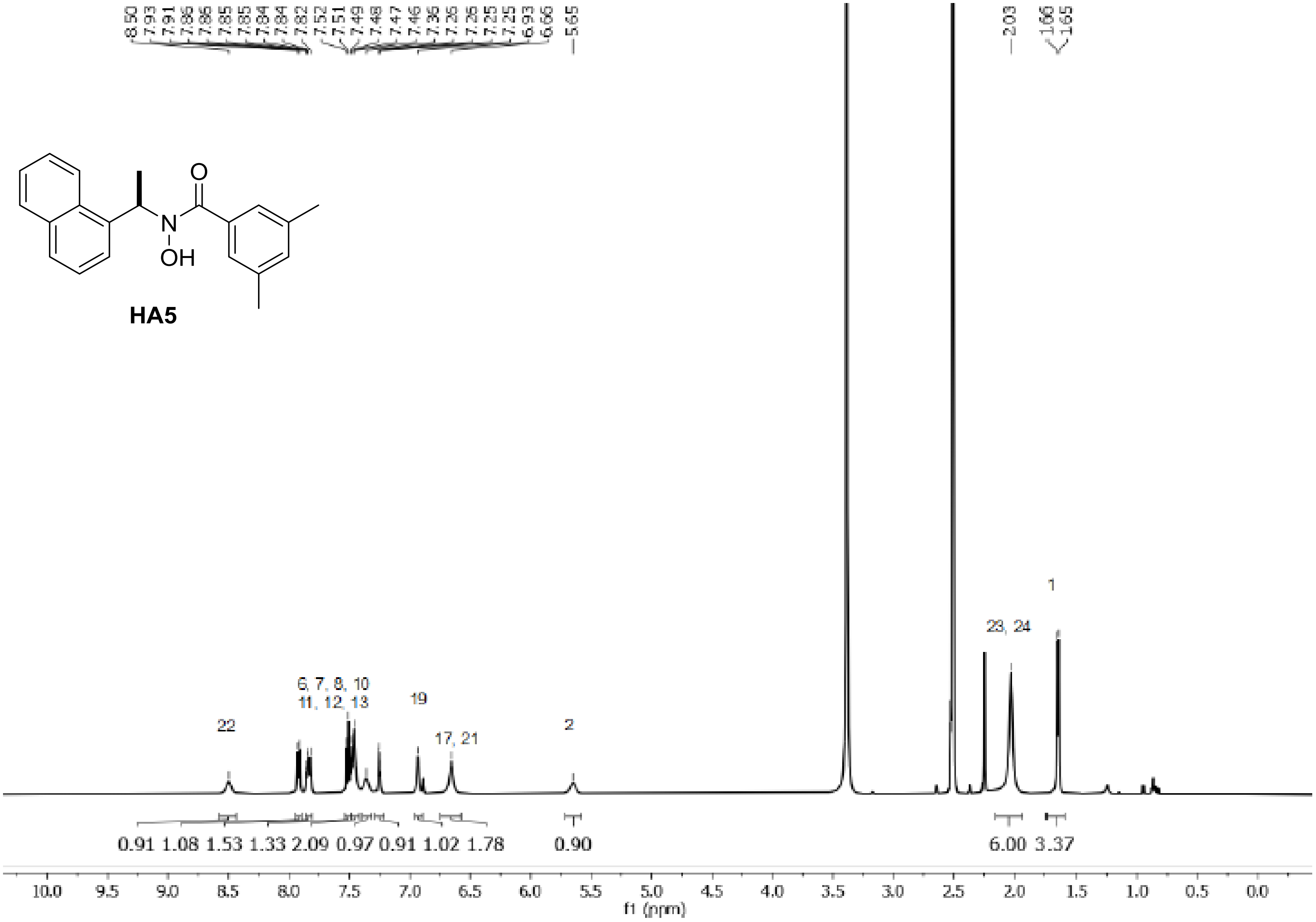

DEPTQ135 NMR Spectrum (500 MHz, DMSO-d_6, 25 °C) of HA5

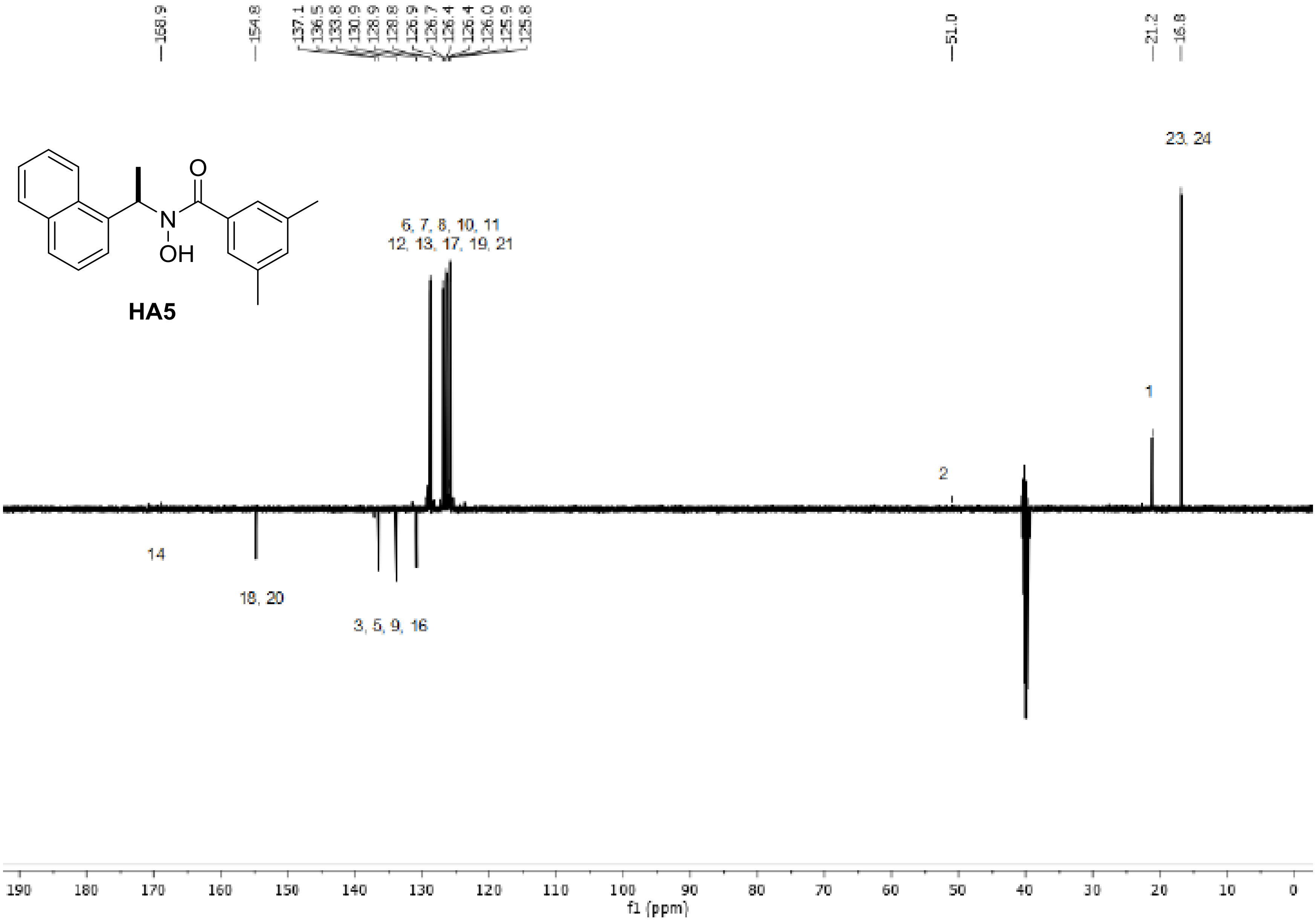

^{1}H NMR Spectrum (500 MHz, DMSO-d_6, 50 °C) of HA6

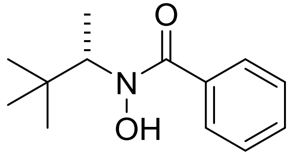

HA6

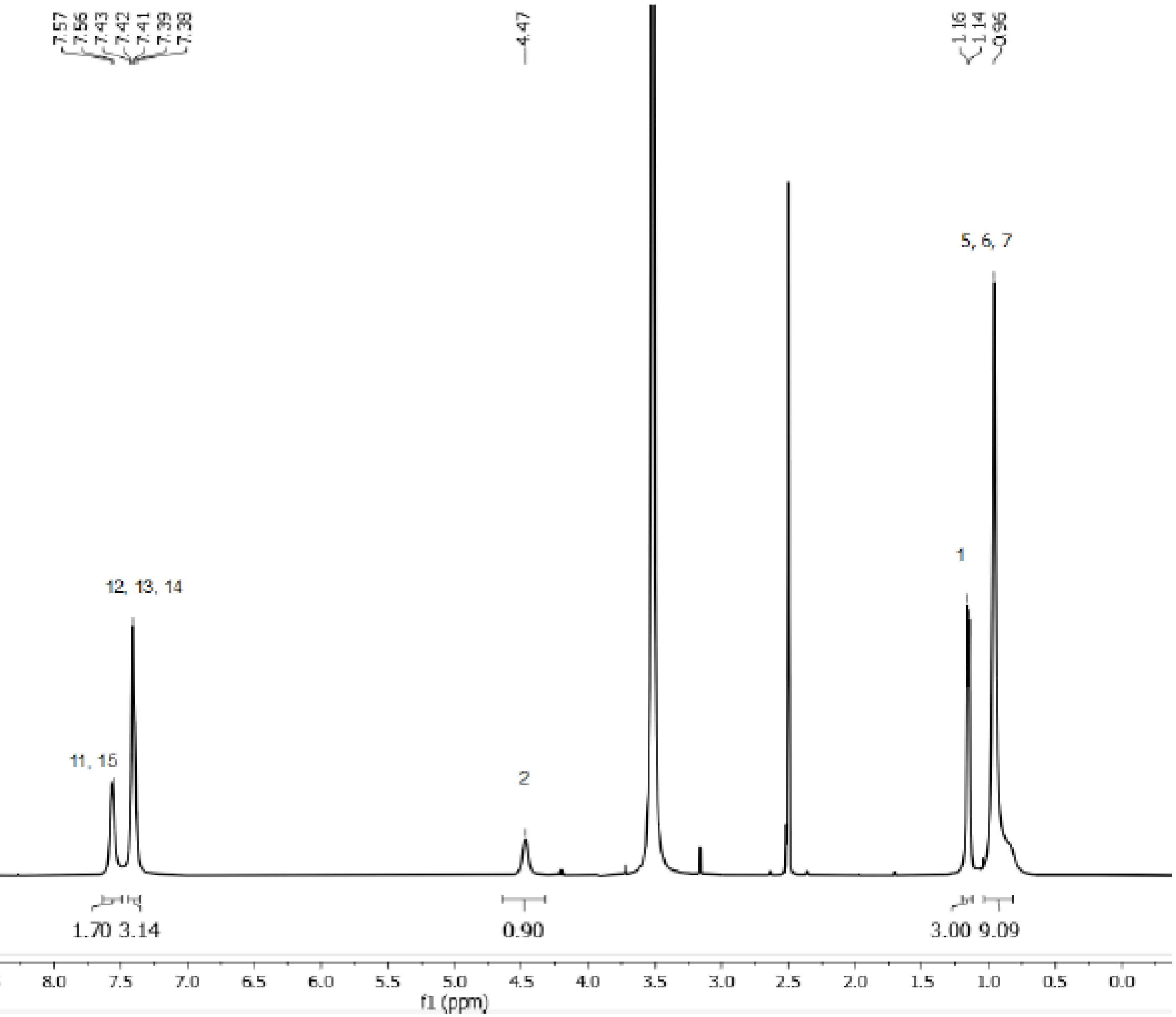

DEPTQ135 NMR Spectrum (500 MHz, DMSO-d_6, 50 °C) of HA6

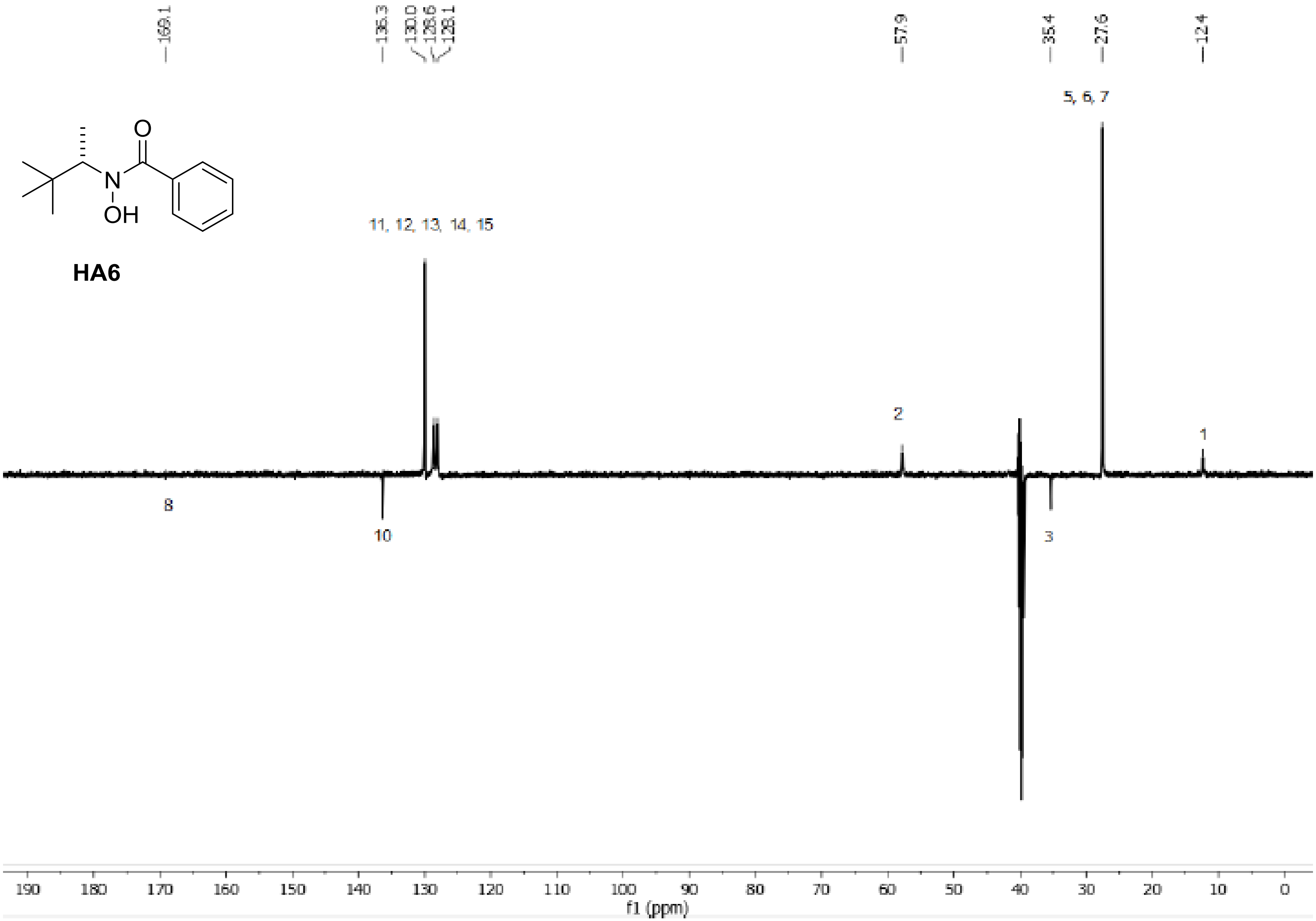

^{1}H NMR Spectrum (500 MHz, DMSO-d_6, 50 °C) of HA7

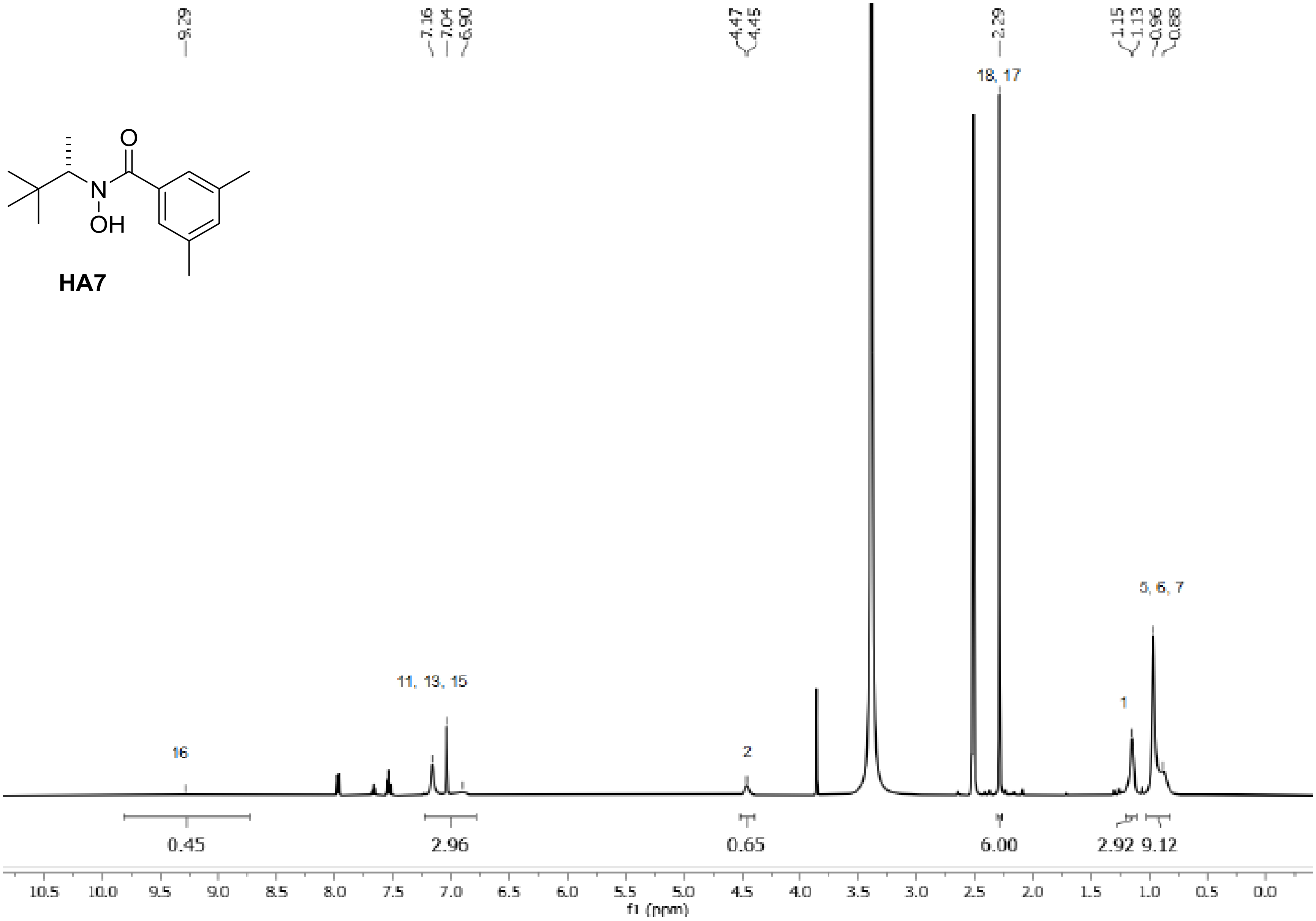

DEPTQ135 NMR Spectrum (500 MHz, DMSO-d_6, 50 °C) of HA7

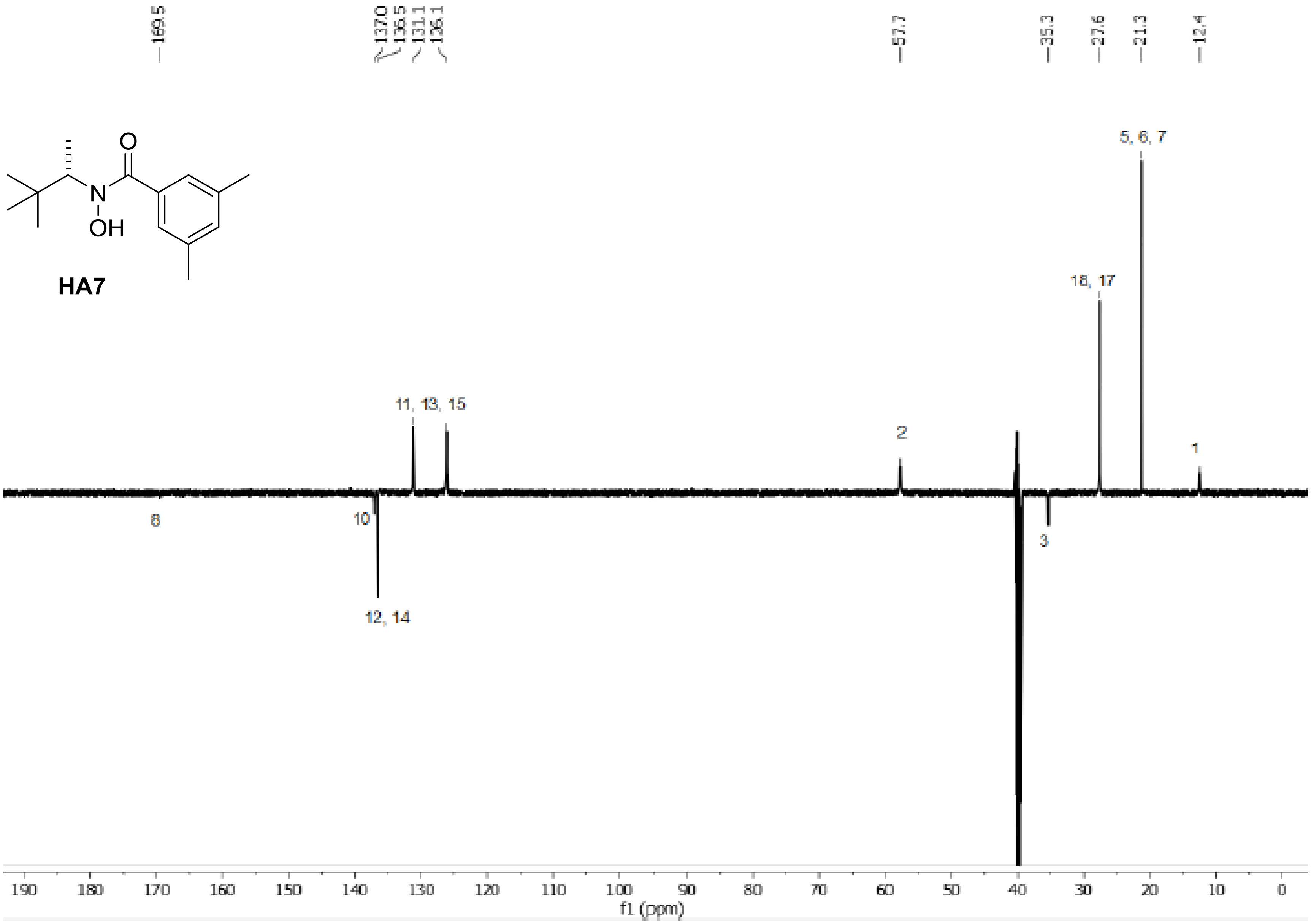

Supplement: Supplementary file 1 [file molecules-30-04311-s001.zip › molecules-3959912-supplementary.pdf]
